# Ionic liquid electrolyte selection for high voltage supercapacitors in high-temperature applications

**DOI:** 10.3389/fchem.2024.1349864

**Published:** 2024-03-04

**Authors:** Ahmed Bahaa, Ayoob Alhammadi, Kallidanthiyil Chellappan Lethesh, Rahmat Agung Susantyoko, Musbaudeen O. Bamgbopa

**Affiliations:** R&D Centre, Dubai Electricity and Water Authority (DEWA), Dubai, United Arab Emirates

**Keywords:** ionic liquid, supercapacitor, high voltage, high temperature, electrolyte screening, electrolyte selection

## Abstract

Systematic analyses of electrolyte physicochemical properties are important to screen ionic liquids (ILs) and understand the electrochemical performance of supercapacitor electrolytes. This study harmonizes the evaluation of electrochemical performance and transport properties of eight shortlisted ILs from 22 commercially available hydrophobic ILs toward achieving a ≥ 5 V supercapacitor capable of high-temperature operation (up to 353.15 K). The eight ILs are N-Propyl-N-methylpyrrolidinium bis(trifluoromethanesulfonyl)imide ([Pyr _1, 3_] [TFSI], N-Pentyl-N-methylpyrrolidinium bis(trifluoromethanesulfonyl)imide ([Pyr _1, 5_] [TFSI]), N-Propyl-N-methylpyrrolidinium bis(fluorosulfonyl)imide ([Pyr _1, 3_] [FSI]), 1-Methyl-1-(2-methoxyethyl)pyrrolidinium Bis(trifluoromethanesulfonyl)imide ([Pyr _1, 102_] [TFSI]), 1-Methyl-1-propylpiperidinium bis(trifluoromethanesulfonyl)imide ([Pip _1, 3_] [TFSI]), 1-Methyl-1-propylpiperidinium bis(fluorosulfonyl)imide ([Pip _1, 3_] [FSI]), N-Trimethyl-N-propylammonium bis(trifluoromethanesulfonyl)imide ([N _111, 3_] [TFSI]), N-Trimethyl-N-hexylammonium bis(trifluoromethanesulfonyl)imide ([N _111, 6_] [TFSI]). The density, viscosity, and ionic conductivity of the eight ILs were measured between 278.15 and 373.15 K to confirm the effects of temperature and ion structure before electrochemical characterization. The [FSI]-based ILs ([Pip _1, 3_] [FSI] and [Pyr _1, 3_] [FSI]) showed lower densities and viscosities compared to other ILs among the eight based on [TFSI]. Consequently, the highest conductivity was obtained for [Pyr _1, 3_] [FSI]. Cyclic voltammetry and impedance spectroscopy was performed on supercapacitors assembled with the eight ILs as electrolytes between 298.15–353.15 K. Conclusion from the two-electrode supercapacitors using multi-walled carbon nanotubes showed the 6 most-applicable ILs towards the targeted ≥ 5 V SC at high temperature are [Pip _1, 3_] [TFSI] (5.4 V), [Pip _1, 3_] [FSI] (5 V), [N _111, 3_] [TFSI] (5.1 V), [N _111, 6_] [TFSI] (5.2 V), [Pyr _1, 102_] [TFSI] (5.2 V), and [Pyr _1, 5_] [TFSI] (5.2 V).

## 1 Introduction

Supercapacitors (SCs) have gained much research attention for energy storage because of their fast charging and discharging, high cycle life, safe operation, and high power density. (Bahaa et al., 2019; Naseri et al., 2022; Pershaanaa et al., 2022). The efforts to increase SC energy density continue due to voltage range limitations in aqueous electrolytes. The limited voltage limits SC technology applications even though they have several intriguing properties. (Balamurugan et al., 2018; Bahaa et al., 2022a). Undesired reactions like hydrogen and oxygen evolution in SCs with aqueous alkali and acidic electrolytes hinder potential windows and cause terminal degradation. (Muzaffar et al., 2019; Guo et al., 2022). Therefore, other electrolytes should be considered to solve the potential window limitations toward increasing energy density and capacitance of assembled SCs. (Qi et al., 2017; Bahaa et al., 2022b).

The application of ionic liquids (ILs), especially room temperature ILs, is increasing in different fields such as energy storage, (Eftekhari, 2017; Qi et al., 2019; Lethesh et al., 2021), liquid-liquid extraction (Shah et al., 2016; Marsousi et al., 2019; Phakoukaki et al., 2022), and biomass processing. (Halder et al., 2019; Jing et al., 2021; Achinivu et al., 2022). Due to their design flexibility and intrinsic mobility of ions, ionic liquids (ILs) are promising for electrochemistry. (Eftekhari, 2017). ILs, like molten salts, are a mobile ion matrix rather than a solvent, at least in electrochemistry. (Eftekhari et al., 2016). The high electrochemical stability window of many ILs provides a platform for designing electrochemical energy storage systems with high-energy densities–making them good candidates for supercapacitors, particularly those based on double-layer charging. (Haque et al., 2021; Miao et al., 2021).

ILs are made up of cation-anion pairs, and the pairing allows tuning of their physicochemical properties to the application. (Giernoth, 2010; Nasir Shah et al., 2022). A systematic study of both physicochemical and transport properties of commercially available ILs helps researchers select suitable ILs for their applications, thereby speeding up product development and improving process economics. (Shah et al., 2015; Lethesh et al., 2019). Experimental data from such systematic studies also helps improve the accuracy of the property prediction models, which can subsequently be applied in IL process design and development. (Venkatraman and Lethesh, 2019). A peculiar characteristic of ILs, which often complicates property measurements, is that their physicochemical properties can significantly vary with the impurities present, especially when novel ILs are to be developed/synthesized in-house. (Freire et al., 2007). As reported earlier, (Seddon et al., 2000), impurities such as water and halides can significantly affect the properties of ILs and distort measurements. Purifying ILs is tedious and cumbersome, increasing the price of ILs while making their large-scale applications economically less viable. (Chakrabarti et al., 2014; Mourad et al., 2017). Admittedly, the properties of ILs also largely depend on the nature of the cations and anions used. (Jeong et al., 2019). A further understanding of their physicochemical properties is necessary to select a suitable option from commercially available ILs for a particular application, process, or device design. (Stettner and Balducci, 2021; Wang et al., 2022). Mass and ionic transport is an important consideration for high-performing and safe-energy storage systems applying ILs. Therefore, targeting high ionic conductivity with reasonable transport properties helps avoid side reactions that can cause degradations to shorten cycle life. (Wang et al., 2014). Additionally, transport properties influence the practical electrochemical stability window and the overall energy density of the assembled device.

**FIGURE 1:**
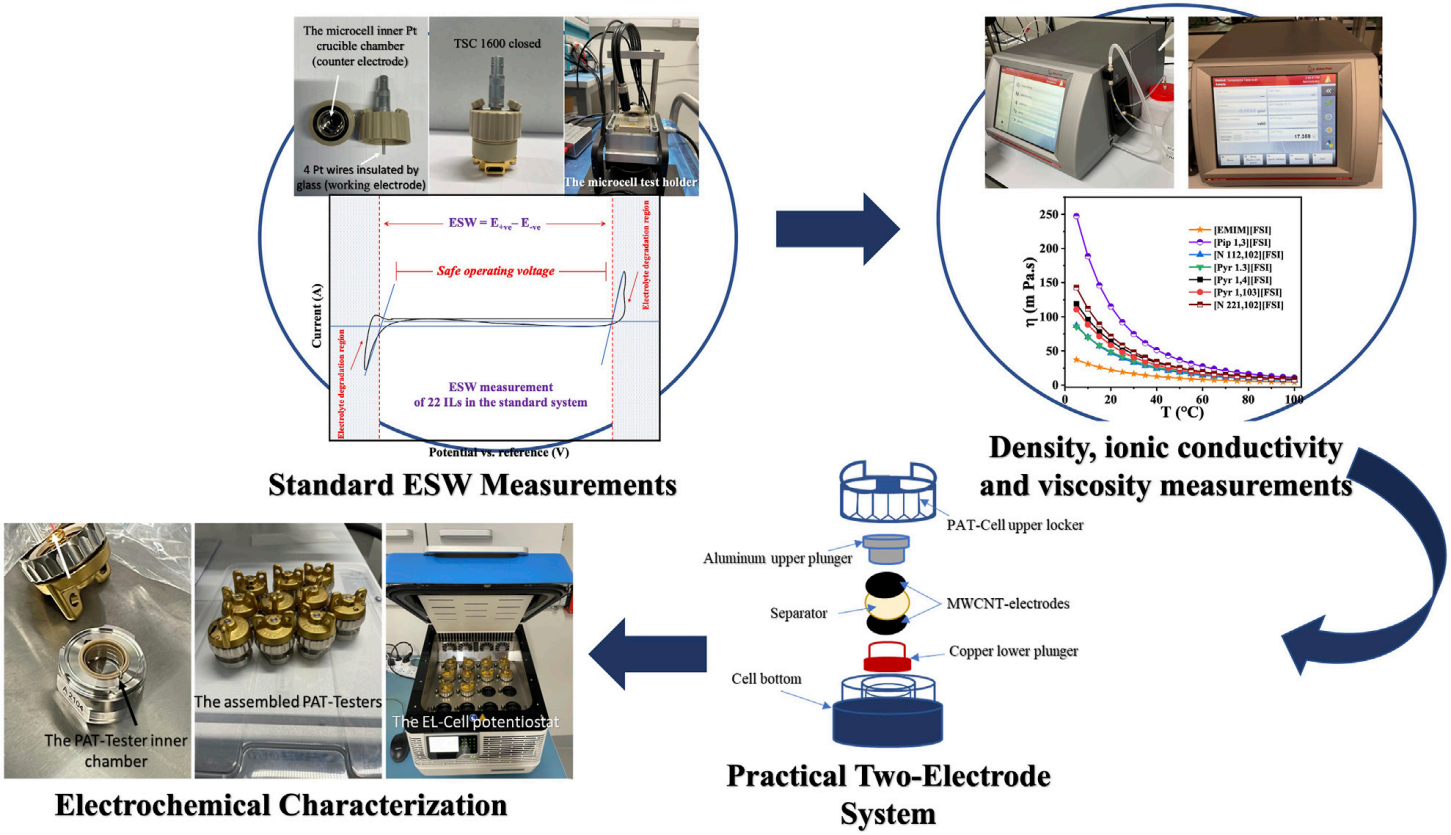
Schematic of the IL screening process for the ≥5 V supercapacitor at elevated temperatures.

This study harmonizes the evaluation of both electrochemical and transport properties of shortlisted ILs from 22 commercially available ILs as design considerations toward achieving a ≥5V supercapacitor capable of high-temperature operation (up to 353.15 K). Our design process for this purpose, consistent with state-of-art (Anouti et al., 2008; Lin et al., 2011; Timperman et al., 2012), is summarized in the schematic of Figure 1. We follow the target of 5 V based on detailed electrochemical stability window evaluations of the same 22 ILs reported in our recent study. (Chellappan et al., 2022). Herein, observations from the physicochemical property measurements are subsequently used to shortlist eight ILs. The list is then narrowed to the most promising electrolyte systems after they are tested as electrolytes for high-voltage supercapacitors at elevated temperatures (up to 80°C), applying a practical electrode material.

## 2 Experimental

### 2.1 Chemicals

The names, abbreviations, and CAS registry numbers of the ILs evaluated in this study are given in Supplementary Table S1. All 22 ILs were purchased at 99.9 % purity from *Solvionic* (France) and obtained in Argon-packed containers. All the ILs were used as received without additional purification. The water content in the ILs is less than 20 ppm—as per the material safety data sheet from the supplier. Multi-walled carbon nanotubes (MWCNTs) were purchased from *Applied Nano-Structured Solutions* (USA). (Susantyoko et al., 2017)

### 2.2 Density and viscosity measurements

The densities and viscosities of the ILs were measured at atmospheric pressure using *SVM 3001* density and viscosity meter (*Anton Par*, Germany)—calibrated with its accompanying reference standards. Densities (*ρ*) and dynamic viscosities (*η*) of the ILs were measured from 278.15 to 373.15 K. Standard measurement uncertainties are u(*ρ*) = ± 0.00001 g cm^−3^, u(*η*) = ± 0.32% mPa.s, and u(*T*) = ± 0.01 K.

### 2.3 Ionic conductivity measurement

Electrochemical impedance spectroscopy (EIS) was applied to determine the dc-ion conductivity in temperatures ranging from 278.15 to 353.15 K. Details of the measurement setup and process are summarized as follows (see illustration in Supplementary Figure S1).

A *TSC 1600* closed measuring cell attached to a *Microcell HC* setup (*RHD instruments* GmbH and Co. KG) was used to measure the sample’s impedance. Sealed IL samples were opened inside an Argon-filled glove box (*MBRAUN*, Germany) at H_2_O and O_2_ concentrations <0.1 ppm. For each experiment, 1.2 mL of the respective IL samples were deposited into the cells. The three-electrode cell sets up a platinum counter electrode with its crucible, while a 0.25 mm-diameter platinum wire isolated by the glass is the working electrode. AgCl-coated Ag wire was used as a pseudo-reference. This reference’s stability was analyzed in our previous work (Chellappan et al., 2022), which shows that the established Ag/Ag^+^ quasi-reference is −0.355 V vs. Fc/Fc^+^ at 298.15 K—with a temperature coefficient of approximately 0.65 mV/K. The airtight measurement cell was moved outside the glovebox for EIS characterization with an *AutoLab PGSTAT302N* Potentiostat (*Metrohm*, Germany).

The EIS experiment was done according to manufacturer-supplied application notes (Falk, 2013) for dc-ion conductivity measurement. Impedance spectra of the samples were obtained at set temperatures with 10 mV voltage amplitude in a frequency range of 150 kHz–5 kHz. Subsequent equivalent circuit analysis of the EIS spectra fit was performed following the application note, ensuring negligible shifts of process-related time constants. The modified Randle’s equivalent circuit is seen in Supplementary Figure S1 where total resistance (*R*_s_ + *R*_ct_) represents the resistance across the IL. The dc-ion conductivity for the ILs at each temperature is then obtained as the product of (*R*_s_ + *R*_ct_) and a cell constant (15.7 cm^−1^). Temperature increment of the setup was done gradually in five steps (from 278.15 to 353.15 K), with a 10-min waiting time between steps for temperature equilibration.

### 2.4 Two—electrode electrochemical characterization

An *EL-Cell PAT-Tester-i-16* (GmbH-Germany) was utilized for cyclic voltammetry (CV) and EIS in two-electrode configurations. Aluminum and copper plungers were used as positive and negative electrode supports/current collectors. Custom MWCNT electrodes of 1.6 cm diameter, whose fabrication is described in our earlier work, (Susantyoko et al., 2017), were used as working electrodes on both sides. The electrode preparation is summarized thus: MWCNT flakes were mixed with 200 mL DI-water: ethanol solution (in 2:1), while the mixture was exfoliated for 10 min using tip sonication. The resulting slurry was cast on a copper foil sheet, and the final MWCNTs casted sheet was dried at 100°C for 1 h before use.

The separator for the two-electrode supercapacitor arrangement was a Whatman GF/A microfiber filter (260 µm thickness), while PEEK-type insulation sleeves were used for precise concentric alignment of the cell arrangement for high-temperature purposes. All electrolytes were handled in an Argon-filled glovebox, while the closed, completely air/water-secluded cell assemblies were transferred outside the glovebox for electrochemical testing. The CV tests were performed at a scan rate of 20 mV/s between 298.15–353.15 K, while two-electrode EIS was performed with 10 mV amplitude between 100 kHz—0.1 Hz in the same temperature range. Each cell arrangement had 120 µL of the respective IL electrolytes for the two-electrode characterizations.

## 3 Results and discussion

### 3.1 Electrochemical stability and density measurements

Given our supercapacitor’s 5 V design goal, a recap of the electrochemical stability window (*ESW*) measurements from our earlier work (see Supplementary Table S1) shows that 15 of the 22 commercial ILs satisfy the criteria, albeit with the measurements on standard platinum electrodes. The tangent fits around the CV vertex potentials technique have been applied (Mousavi et al., 2015) to determine the *ESW*s. This technique accounts for the impacts of mass transfer in the electrolyte bulk, the electrode matrix (for porous electrodes), and the electrolyte-electrode interface to provide a more realistic quantification of electrochemical stability. These mass transfer considerations dictate the effective ionic diffusivity in the system, which is proportional to the slopes of the tangent fits. (Mousavi et al., 2015; Bard et al., 2022). All else being the same, an increasing tangent slope shows lower effective ion diffusivity, while lower tangent slopes indicate higher mass transport constraints on diffusivity, as shown in Supplementary Figure S2. Supplementary Figure S2 also depicts the real anodic and cathodic potential limits of the tested ILs in the standard system, which helped us select the optimum ILs for the practical system. Based on this method, eight ILs, which show the best mass transport characteristics and could be operated safely from low to high temperatures (among the 15 with >5 V ESW), have been selected here in this study. The eight most promising ILs are [Pyr _1, 3_] [TFSI], [Pyr _1, 3_] [FSI], [Pyr _1, 5_] [TFSI], [Pyr _1, 102_] [TFSI], [Pip _1, 3_] [TFSI], [Pip _1, 3_] [FSI], [N _111, 3_] [TFSI], and [N _111, 6_] [TFSI]. These ILs include pyrrolidinium, piperidinium, and tetraalkylammonium cations combined with either bis(trifluoromethane sulfonyl) imide (TFSI) and bis(flurosulfonyl)imide (FSI) anions (see Supplementary Table S1). Another rationale behind the initial 22 commercial ILs screened was their hydrophobicity, relatively low viscosity, and reasonably good conductivity, making them good candidates for electrochemical applications.

**FIGURE 2:**
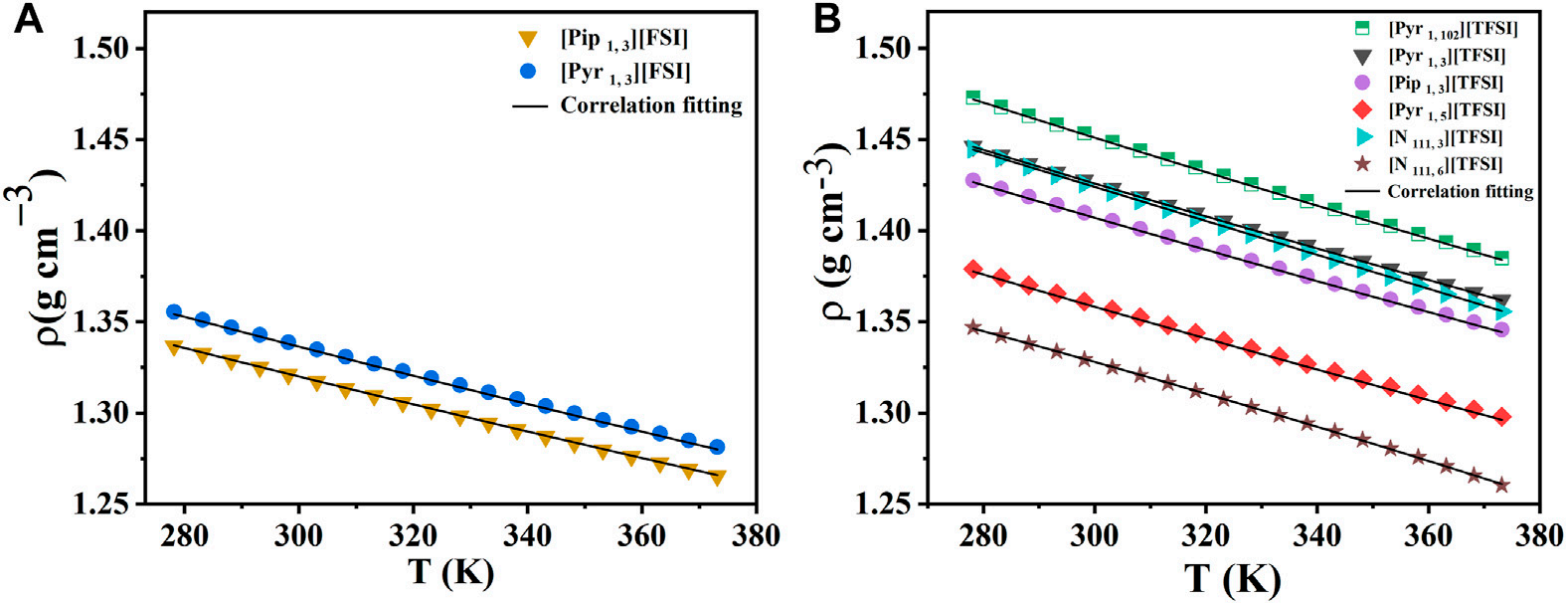
Density measurements with temperature for the eight shortlisted ILs. **(A)** [FSI] anion-based. **(B)** [TFSI] anion-based. Markers represent experimental data. Lines represent the correlation fits of density as a function of temperature (*T*) using: $\rho =A+BT+CT^{2}$ , where correlation coefficients are *A* (g cm^-3^), *B* (g cm^-3^ K^−1^), and *C* (g cm^-3^ K^−2^). See Supplementary Material for details and reference of curve fits.

The density of ILs mainly depends on cation-anion interactions and the packing of molecules. In general, the nature of the IL anions strongly influences density. To further corroborate our mass transport discussions, we measured the density of the ILs in the same temperature range of 278.15 K–373.15 K. The measurement results are presented in Figures 2A, B. See Supplementary Tables S2 and S3 for the data.

As expected, the density of all ILs measured decreased linearly with increased temperature (in K). Among the ILs studied, [N _111, 6_] [TFSI] and [Pip _1, 3_] [TFSI] showed the lowest density values at 298.15 K (1.3293 and 1.3297 g cm^−3^, respectively). This observation, particularly for [N _111, 6_] [TFSI], was consistent with earlier reports that an increase in the alkyl spacer length can decrease the density of ILs. (Lethesh et al., 2014). This might be because of the compression in the aliphatic region due to the van der Waals forces, which will affect the free volume of the ILs. (Shah et al., 2015). This trend was also observed in the case of pyrrolidinium-based ILs. For instance, the density of [Pyr _1, 3_] [TFSI] is 1.4275 g cm^−3^ at 298.15 K, which is reduced to 1.3611 g cm^−3^ when the alkyl spacer length increased to five carbon atoms for [Pyr _1, 5_] [TFSI]. [Pyr _1, 102_] [TFSI] displayed the highest density (1.4533 g cm^−3^) at a similar experimental condition, which is in agreement with the earlier reports that shorter ether groups make increased-density ILs. (Raj et al., 2017). Among the two anions studied, [FSI] anion-based ILs generally showed a lower density than [TFSI] anions, as seen in Figure 2. The lower density for [FSI] anion-based ILs may be because of the smaller size of the [FSI] anion compared to [TFSI] anion. The effect of relative ion sizes was also observed when comparing the density of pyrrolidinium and piperidinium-based ILs.

Our density measurements for some select ILs were compared with available literature-reported values in Supplementary Table S4, further validating the reproducibility of our experimental data and procedure. While the earlier reported density data were performed at room temperature, the respective ILs have been tested in a wide operating temperature range, opening the pathway for practical applications at extreme temperatures. Practical applications like redox-flow or hybrid batteries often require fluid flow properties of the electrolyte, like density. In addition, packing and sealing supercapacitors for high-temperature operation also require known fluid flow properties.

### 3.2 Dynamic viscosity and ionic conductivity

**FIGURE 3:**
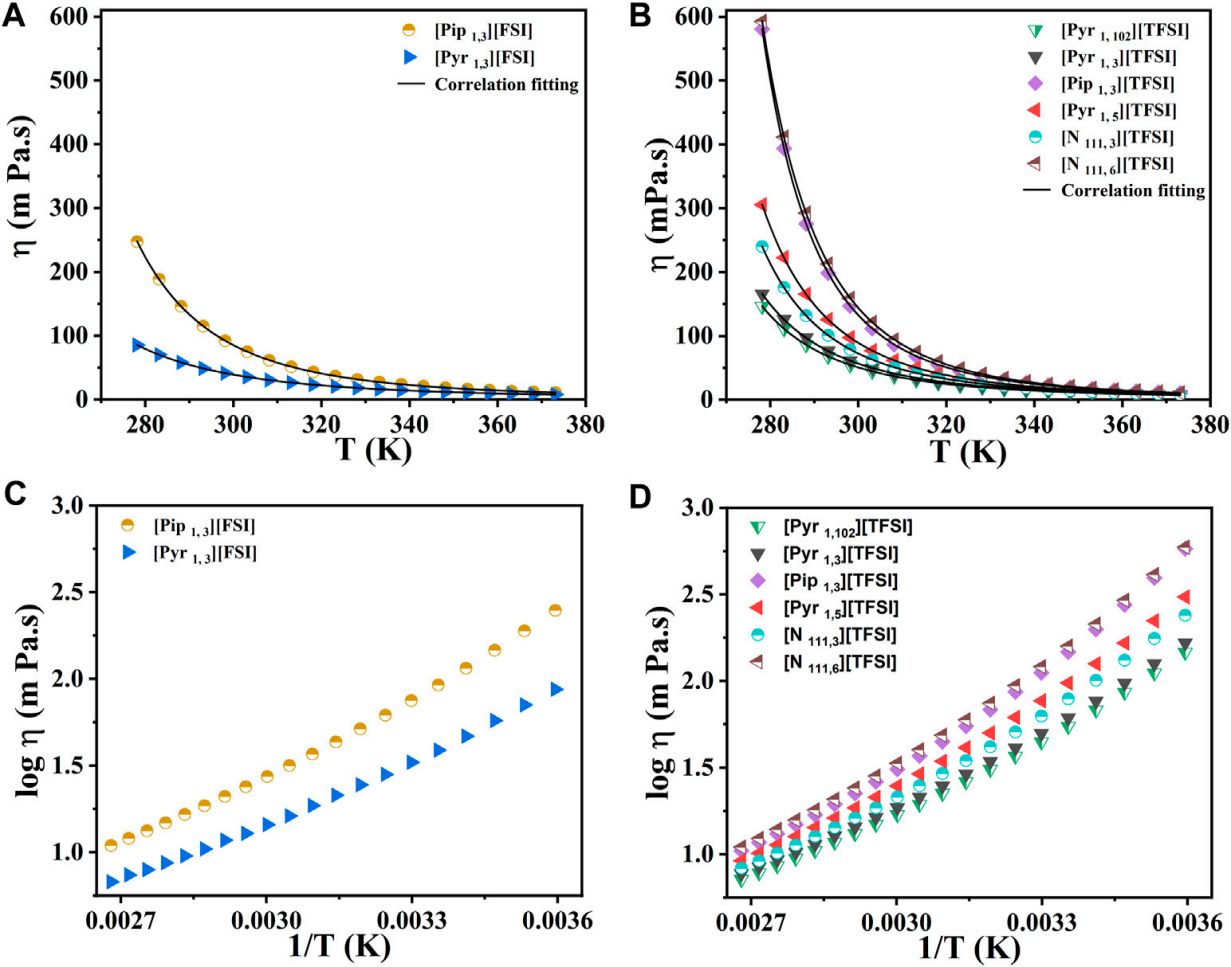
The dynamic viscosity measurements with temperature for the eight shortlisted ILs. **(A,C)** [FSI] anion-based. **(B,D)** [TFSI] anion-based. Markers represent experimental data points. Lines in **(A)** and **(B)** represent the correlation fits of viscosity as a function of temperature (*T*) using the classical Vogel−Fulcher−Tammann (VFT) equation: $\eta =\eta_{0\;}\exp\;\left(D/\left(T\;-\;T_{0}\right)\right)$ . Where correlation coefficients are $\eta_{0}$ (mPa.s), *D* (K), and $T_{0}$ (K). See the Supplementary Material for details and reference of curve fits.

The dynamic viscosities of the eight shortlisted ILs were measured between 278.15 K and 373.15 K. The measured viscosity values shown in Figures 3A–D for the eight ILs range from 41.36 mPa s to 158.75 mPa s at 298.15 K. A quick survey indicates that our dynamic viscosity results also agree with the literature (see Supplementary Table S4). The numerical values of results in Figure 3 are also tabulated in the (Supplementary Tables S5 and S6). At 298.15 K, the lowest viscosity was observed for [Pyr _1, 3_] [FSI] (41.36 mPa s), while [N _111, 6_] [TFSI] showed the highest viscosity of 158.75 mPa s at 298.15 K. In the case of pyrrolidinium-based ILs, the increase in the alkyl chain length on the cation led to increased viscosity.

For instance, the viscosity increased from 61.19 mPa s (for [Pyr _1, 3_] [TFSI]) to 97.38 mPa s at 298.15 K when the alkyl spacer length increased to five carbon atoms to make [Pyr _1, 5_] [TFSI]. This increase in viscosity for the pyrrolidinium-based ILs was attributed to the higher Van der Waals interaction between the longer alkyl groups. (Schrekker et al., 2008; Filippov et al., 2014). A similar trend was observed for tetra alkyl ammonium-based ILs at similar experimental conditions, where [N _111, 3_] [TFSI] reported 78.92 mPa s. The ether-functionalized ILs showed lower viscosity compared to their alkyl analogs. For example, the [Pyr _1, 102_] [TFSI] showed lower viscosity (54.61 mPa s) compared to [Pyr _1, 3_] [TFSI] (61.19 mPa s) and [Pyr _1, 5_] [TFSI] (97.38 mPa s) at 298.15 K, which is related to the flexibility in rotation offered by the oxygen atom on the ether functionality. (Fleshman and Mauro, 2019).

**FIGURE 4:**
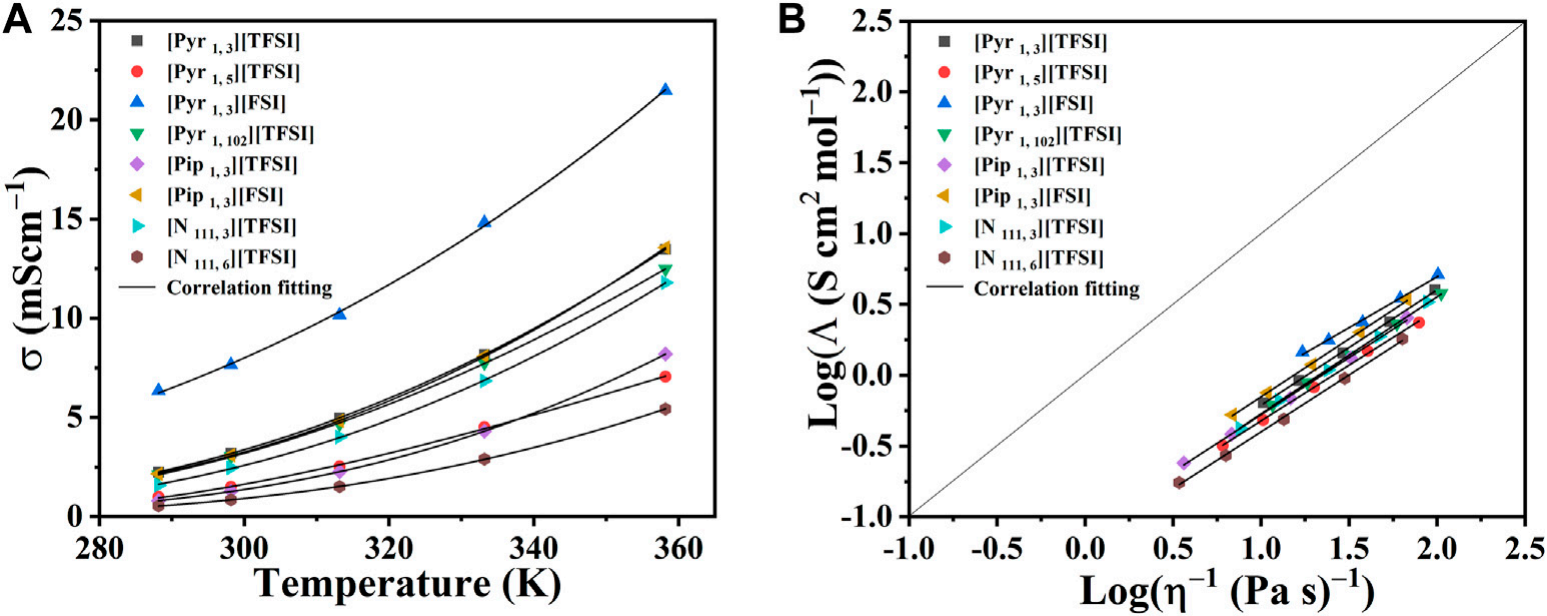
**(A)** Dc-ionic conductivity of the eight shortlisted ILs at temperatures from 278.15 K to 358.15 K in a three-electrode system. Markers represent experimental data points. Lines represent the correlation fits of ionic conductivity as a function of temperature (*T*) using the equation: $\sigma =\sigma_{0}\exp\left((\frac{E}{\left(T_{0}-T\right)}\right))$ , where correlation coefficients are $\sigma_{0}$ (mS cm^-1^), *E* (K), and $T_{0}$ (K). **(B)** Walden plots of molar conductivity, Λ $\left(\sigma \times Molar\;mass/Density\right)$ , and viscosity η for ILs. The experimental points were fitted using: log Λ = log C′ + α log η^−1^. See Supplementary Material for details and reference of curve fits.

As expected, a significant decrease in the viscosity of all the ILs under investigation was observed at elevated temperatures (up to 373.15 K). The results showed that at high temperatures, the viscosities of the eight ILs became more similar, which is an advantage in improved mass transport. The dc-ionic conductivity measurements (from 278.15 K to 358.15 K) for the eight ILs, [Pyr _1, 3_] [TFSI], [Pyr _1, 5_] [TFSI], [Pyr _1, 3_] [FSI], [Pyr _1, 102_] [TFSI], [Pip _1, 3_] [TFSI], [Pip _1, 3_] [FSI], [N _111, 3_] [TFSI], and [N _111, 6_] [TFSI], are presented in Figure 4A (see data in Supplementary Table S7). Ionic conductivity and viscosity of IL electrolytes are linked and crucial since they directly affect their ability to serve as charge carriers in energy storage applications. Higher viscosities result in lower ionic conductivities and *vice versa*. Therefore, Figure 4B relates molar conductivity to viscosity and also shows the deviation of measurements to the ideal Walden plot line. The link between conductivity and viscosity references ionic mobility/diffusivity under current flow being affected by viscosity. (Watanabe et al., 2017; Kar et al., 2019). The advantage of ILs over organic solvent-based electrolytes at higher temperatures is that ionic conductivity significantly improves (as viscosity drops) in ILs. In addition, there are often safety, vaporization, or precipitation concerns with organic solvents, which is not the case with ILs.

Combining observations in Figure 3, Figure 4A, [Pyr _1, 3_] [FSI] stands out in terms of highest conductivity (≈21.5 mScm^−1^) with the lowest recorded viscosity (≈9.9 mPa s) at a high temperature of 358.15 K. At the other end of the spectrum, [N _111, 6_] [TFSI] showed the lowest conductivities (Figure 4) as it records the highest viscosities (Figure 3) in the measured temperate range.

The other ILs also follow the same expected viscosity and ionic conductivity dependency. Furthermore, [Pyr _1, 3_] [FSI] appears as the most attractive in terms of conductivity and viscosity. The pyrrolidinium cation-based ILs in the shortlist also appear generally adequate, while [Pip _1, 3_] [FSI] and [N _111, 3_] [TFSI] are also worthy of secondary attention in terms of conductivity and viscosity. The impact of alkyl chain lengths plays a role in the pyrrolidinium cation-based ILs.

### 3.3 Two-electrode cell electrochemical performance

CV and EIS were conducted in a two-electrode setup using a typical carbon-based electrode material to validate the electrochemical stability findings from Pt electrodes and the transport parameters discussed in previous sections of this work. As observed in this study, there was no significant correlation between high ionic conductivity and observed ESW on standard Pt electrodes (Section 3.1). When typical porous carbon electrode materials are involved, the practical ESW available for meaningful charge storage depends on many factors governing the cell’s ion-ion, ion-bulk electrolyte, bulk electrolyte-bulk electrode, and ion-electrode interface interactions. (Chen et al., 2013). With CV measurements, we can at least qualitatively observe the outcomes of these interactions, determine the charge transport at the electrode/electrolyte interface, and subsequently quantify charge storage.

The eight shortlisted ILs are subsequently adopted to assemble symmetric supercapacitor systems with two MWCNTs-based electrodes. Here, each cation family among the eight shortlisted ILs has been analyzed accordingly. EIS was performed before and after the CV tests to monitor possible changes within the system. The CV tests were performed with increasing upper vertex potential steps to maintain the integrity of the system (given the possibility of irreversible changes close to potential limits).

**FIGURE 5:**
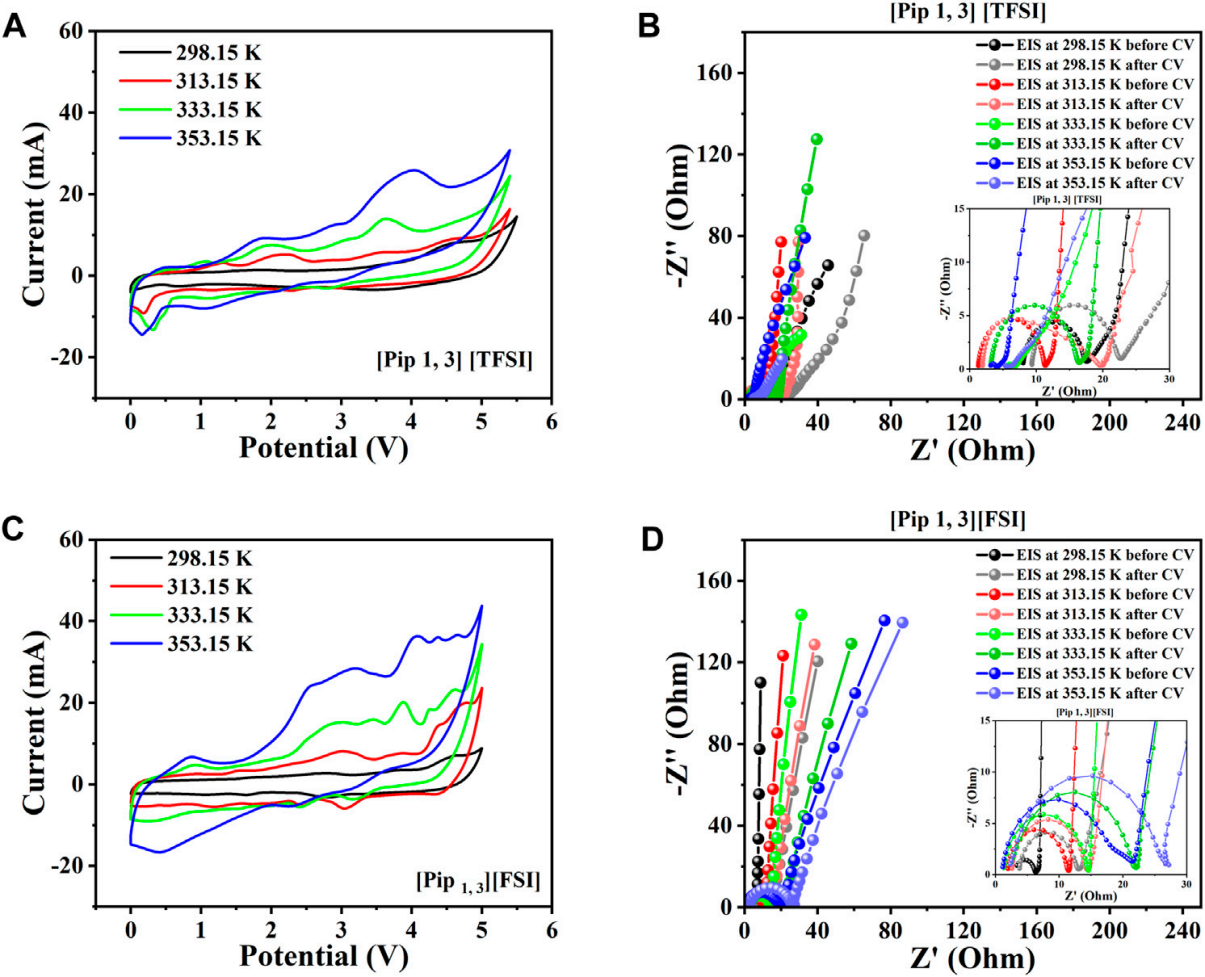
Two-electrode supercapacitor characterization at different temperatures using the piperidinium cation-based ILs among the eight shortlisted ILs. **(A)** Steady-state CV for [Pip _1, 3_] [TFSI]. **(B)** EIS spectra initially before and after CV for [Pip 1, 3] [TFSI]. **(C)** Steady-state CV for [Pip _1, 3_] [FSI]. **(D)** EIS spectra initially before and after CV for [Pip _1, 3_] [FSI].

Figures 5A–D introduce the results from supercapacitors assembled with the piperidinium cations-based ILs among the shortlisted. The figures show the CVs, initial EIS spectra before CV, and EIS spectra after CV for both supercapacitors assembled with [Pip _1, 3_] [TFSI] and [Pip _1, 3_] [FSI] from 298.15 K to 353.15 K.

[Pip _1, 3_] [TFSI] delivered a practical potential window as high as 5.4 V, after which significant degradation occurred when we increased the operating potential to 5.5 V (Figure 5A). The supercapacitor with [Pip _1, 3_] [FSI] within the same temperatures gave a slightly lower potential window of 5 V (Figure 5C). Section 2.3 of this work explains that the obtained EIS spectra have been analyzed using the equivalent circuit model in Supplementary Figure S1. As expected, the EIS results in Figures 5B, D generally show better mass transport with increasing temperature due to decreasing viscosity. Consequently, recorded currents in the CVs also increase with temperature elevation. The equivalent series resistance (*R*_s_) at most temperatures for the supercapacitor with [Pip _1, 3_] [FSI] was lower than that with [Pip _1, 3_] [TFSI] due to the lower viscosity and higher conductivity of [Pip _1, 3_] [FSI], compared to [Pip _1, 3_] [TFSI] (see Figure 3). Obtained charge transfer resistance (*R*_ct_) from the equivalent circuit consistently increased with temperature for the system with [Pip 1, 3] [FSI], which is higher than that with [Pip _1, 3_] [TFSI].

**FIGURE 6:**
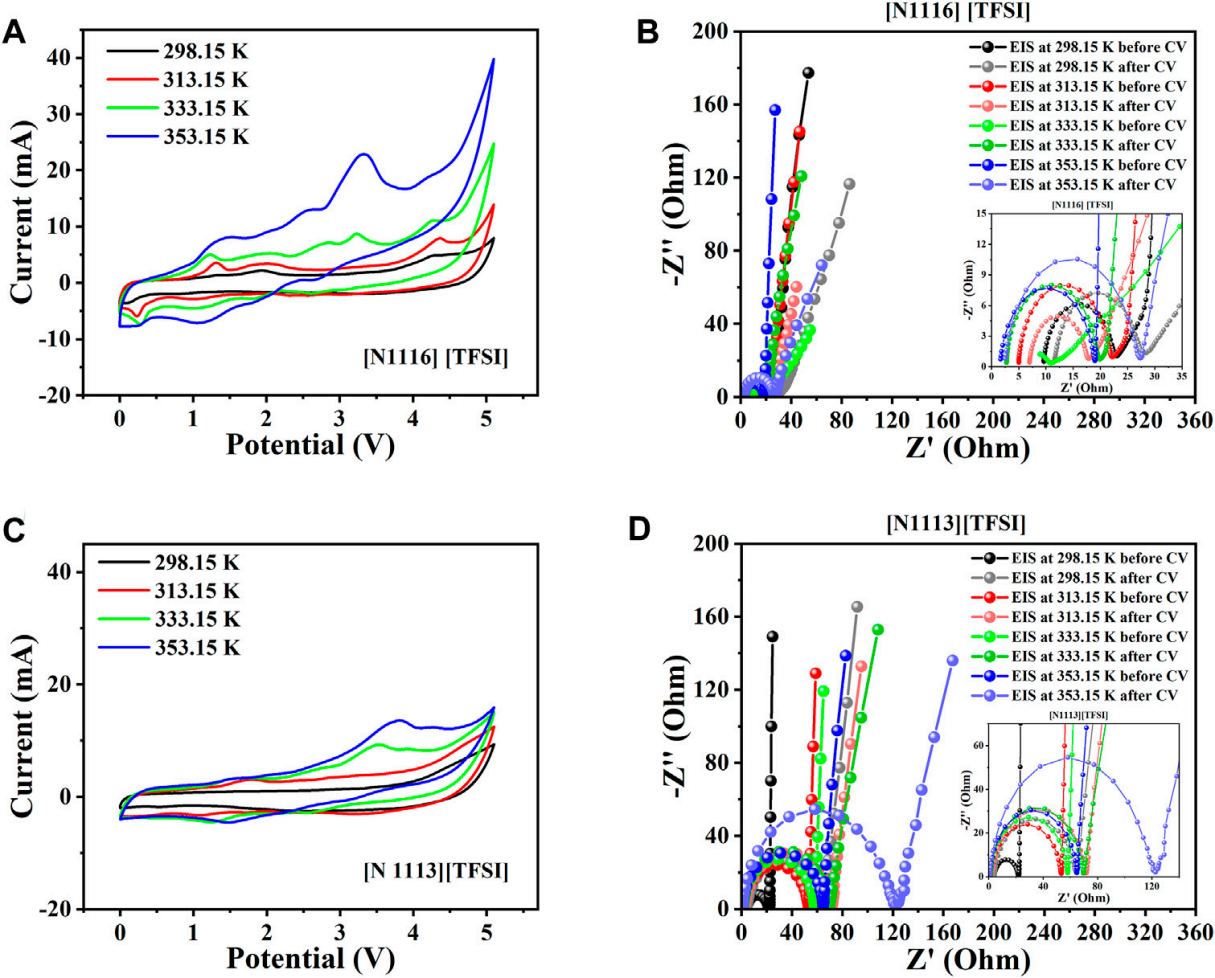
Two-electrode supercapacitor characterization at different temperatures using the tetra-alkyl ammonium cation-based ILs among the eight shortlisted ILs. **(A)** Steady-state CV for [N _111, 6]_ [TFSI]. **(B)** EIS spectra initially before and after CV for [N _111, 6_] [TFSI]. **(C)** Steady-state CV for [N _111, 3_] [TFSI]. **(D)** EIS spectra initially before and after CV for [N _111, 3_] [TFSI].

**FIGURE 7:**
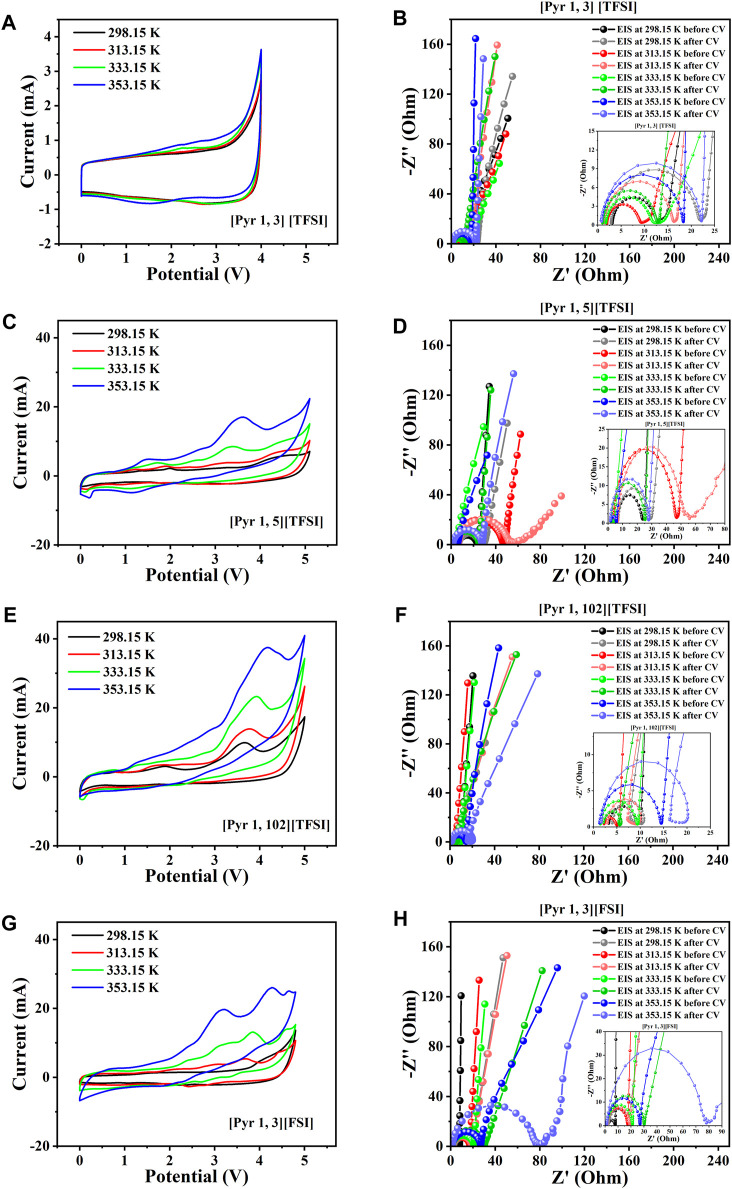
Two-electrode supercapacitor characterization at different temperatures using the pyridinium cation-based ILs among the eight shortlisted ILs. **(A)** Steady-state CV for [Pyr _1, 3_] [TFSI]. **(B)** EIS spectra initially before and after CV for [Pyr _1, 3_] [TFSI]. **(C)** Steady-state CV for [Pyr _1, 5_] [TFSI]. **(D)** EIS spectra initially before and after CV for [Pyr _1, 5_] [TFSI]. **(E)** Steady-state CV for [Pyr _1, 102_] [TFSI]. **(F)** EIS spectra initially before and after CV for [Pyr _1, 102_] [TFSI]. **(G)** Steady-state CV for [Pyr _1, 3_] [FSI]. **(H)** EIS spectra initially before and after CV for [Pyr _1, 3_] [FSI].

Figure 6 compares the CV and EIS results from the supercapacitors assembled with the tetra-alkyl ammonium-based ILs in the shortlist ([N _111, 6_] [TFSI] and [N _111, 3_] [TFSI]). Quite similar potential windows (5.2 V and 5.1 V) were obtained for the systems with [N _111, 6_] [TFSI] and [N _111, 3_] [TFSI, respectively, before degradation became more apparent (see Figures 6A, C). Noticeable enhancement in the conductivity (consequently, *R*_s_) is recorded with increasing temperature for both [N _111, 6_] [TFSI] and [N _111, 3_] [TFSI] systems (Figures 6B, D). However, [N _111, 6_] [TFSI] appears to be more sensitive to temperature than [N _111, 3_] [TFSI]. Figures 7A–H present the CV and EIS results of supercapacitors assembled with the selected pyrrolidinium-based ILs. As with the other systems, *R*_s_ decreases with increasing temperature for the supercapacitors applying pyrrolidinium-based ILs in Figure 7.

Overall, *R*_s_ is expected to decrease with increasing temperatures due to improved conductivity with temperature, as shown earlier for the shortlisted ILs in Figure 4. The nominal values of *R*_s_ with temperature are also expected to show a minor difference in the recorded values before and after the CV due to possible electrode structure reorganization. The EIS analyses were performed before and after the CV tests to monitor significant form distortions in the EIS spectra with increasing temperature, directly indicating significant electrochemical instability or chemical irreversibility. The results so far show a significantly distorted EIS at 353.15 K for the supercapacitor applying [Pip _1, 3_] [TFSI] (Figure 5B).

The supercapacitors with both ammonium cation-based ILs among the eight shortlisted are generally satisfactory, with the [N _111, 6_] [TFSI] system appearing better than the [N _111, 3_] [TFSI] system (Figure 6). However, only supercapacitors applying [Pyr _1, 3_] [FSI] and [Pyr _1, 3_] [TFSI] are satisfactory in terms of electrochemical stability among the pyrrolidinium-based ILs in the shortlist, as seen in Figures 7A–H.

The other pyrrolidinium-based ILs like [Pyr _1, 102_] [TFSI] unfortunately show significantly distorted EIS spectra as temperature increases, especially after the CV (Figures 7E, F). The [Pyr _1, 3_] [FSI] supercapacitor gave a 4.8 V potential window—close to the target. Interestingly, the supercapacitor with [Pyr _1, 3_] [TFSI] recorded consistent CV with increasing temperature, albeit with a more limited potential window of approximately 4 V (Figures 7A, B), which is less than the 5 V design goal. Therefore, considering all discussed observations and the design goal of achieving a ≥ 5 V supercapacitor capable of high-temperature operation (up to 353.15 K). The six most applicable systems to achieve our targeted SC ≥ 5 V are [Pip _1, 3_] [TFSI] (5.4 V), [Pip _1, 3_] [FSI] (5 V), [N _111, 3_] [TFSI] (5.1 V), [N _111, 6_] [TFSI] (5.2 V), [Pyr _1, 102_] [TFSI] (5.2 V), and [Pyr _1, 5_] [TFSI] (5.2 V). Table 1 summarizes the results of the electrochemical performance and characteristic parameters obtained from Figure 5 to Figure 7.

**TABLE 1:** Potential window, equivalent circuit charge transfer, and series resistance (Rct and Rs, respectively) of supercapacitors applying the eight shortlisted ILs at different temperatures were obtained.

T/K	Parameters	[Pyr _1, 3_] [TFSI]	[Pyr _1, 5_] [TFSI]	[Pyr _1, 3_] [FSI]	[Pyr _1, 102_] [TFSI]	[Pip _1, 3_] [TFSI]	[Pip _1, 3_] [FSI]	[N _111, 3_] [TFSI]	[N _111, 6_] [TFSI]
298.15	Potential Window	4 V	5.2 V	4.8 V	5.2 V	5.4 V	5 V	5.1 V	5.2 V
	R_ct_/R_s_—Before CV	13 Ω/3 Ω	17 Ω/6 Ω	8 Ω/3 Ω	9.5 Ω/3 Ω	17 Ω/8 Ω	6.3 Ω/3.4 Ω	22.1 Ω/4.4 Ω	22.6 Ω/9.8 Ω
	R_ct_/R_s_—After CV	22 Ω/3 Ω	29 Ω/6.5 Ω	18.1 Ω/2.9 Ω	10.8 Ω/4 Ω	23 Ω/9 Ω	13 Ω/3.8 Ω	64 Ω/4.2 Ω	27.5 Ω/11.5 Ω
313.15	Potential Window	4 V	5.2 V	4.8 V	5.2 V	5.4 V	5 V	5.1 V	5.2 V
	R_ct_/R_s_—Before CV	9 Ω/2 Ω	48 Ω/3.5 Ω	17.7 Ω/2 Ω	5.2 Ω/2.3 Ω	11 Ω/1 Ω	11.5 Ω/2 Ω	53.2 Ω/2.1 Ω	22.3 Ω/5 Ω
	R_ct_/R_s_—After CV	16 Ω/1.5 Ω	55.5 Ω/6.5 Ω	19.7 Ω/2 Ω	10.1 Ω/2.6 Ω	20 Ω/2 Ω	14.5 Ω/2.6 Ω	71.2 Ω/2.4 Ω	17.5 Ω/7 Ω
333.15	Potential Window	4 V	5.2 V	4.8 V	5.2 V	5.4 V	5 V	5.1 V	5.2 V
	R_ct_/R_s_—Before CV	12.1 Ω/2 Ω	4.8 Ω/2.5 Ω	20.5 Ω/1.6 Ω	9.8 Ω/2 Ω	7 Ω/5 Ω	14.6 Ω/1.2 Ω	57.5 Ω/1.3 Ω	11.5 Ω/7.5 Ω
	R_ct_/R_s_—After CV	12.9 Ω/1.5 Ω	25.2 Ω/2.3 Ω	30.2 Ω/1.5 Ω	6.5 Ω/2.4 Ω	16 Ω/3 Ω	22 Ω/2.3 Ω	69 Ω/1.4 Ω	19.8 Ω/2.6 Ω
353.15	Potential Window	4 V	5.2 V	4.8 V	5.2 V	5.4 V	5 V	5.1 V	5.2 V
	R_ct_/R_s_—Before CV	16.5 Ω/1 Ω	6.1 Ω/2.2 Ω	27 Ω/1.1 Ω	14.8 Ω/1.6 Ω	4 Ω/3 Ω	21.5 Ω/1.1 Ω	64.5 Ω/1 Ω	19.5 Ω/2.3 Ω
	R_ct_/R_s_—After CV	22.4 Ω/1 Ω	28 Ω/2 Ω	79.5 Ω/1.4 Ω	20.5 Ω/1.7 Ω	7 Ω/4 Ω	27.2 Ω/2.3 Ω	122.5 Ω/1 Ω	27.5 Ω/2.5 Ω

## 4 Conclusion

We present an essential systematic analysis of electrochemical and physicochemical aspects to screen commercial ILs as electrolytes for electrochemical applications. Based on prior electrochemical stability window measurements of 22 commercially available ILs and screening, the best candidates to achieve a >5 V supercapacitor capable of high-temperature operation (up to 80 deg. C, or 353.15 K). Using mass transport considerations from CV, we shortlisted the best eight ILs with >5 V stability window ([Pyr _1, 3_] [TFSI], [Pyr _1, 3_] [FSI], [Pyr _1, 5_] [TFSI], [Pyr _1, 102_] [TFSI], [Pip _1, 3_] [TFSI], [Pip _1, 3_] [FSI], [N _111, 3_] [TFSI], and [N _111, 6_] [TFSI]) among the 22. Measurements for density, dynamic viscosity, and ionic conductivity of the chosen eight ILs were performed between 298.15 and 353.15 K to corroborate mass transport. We surmise that practical applications of ILs in electrochemical energy storage (like supercapacitors herein or redox-flow and hybrid batteries) require known physical properties of the electrolyte at different temperatures. As expected, temperature increment significantly reduced viscosity and density for all the ILs, translating to an increase in ionic conductivity. However, the influence of ionic conductivity on electrochemical performance and stability is entirely different when applying standard Pt electrodes or typical porous carbon-based electrodes.

CV and EIS characterization of supercapacitors made with MWCNT electrodes adopting the eight shortlisted hydrophobic ILs (298.15–353.15 K) helped confirm the best ILs to achieve the >5 V for high-temperature application. We believe the systematic screening performed here for commercially available ILs can also be applied to newly synthesized ILs, especially considering electrochemical applications at high temperatures. Furthermore, we consider that possible improvements in the electrode–ILs interactions will vary when novel electrode materials are involved.

## Data Availability

The original contributions presented in the study are included in the article/Supplementary Material, further inquiries can be directed to the corresponding author.

## References

[B1] Achinivu E. C. Cabrera M. Umar A. Yang M. Baral N. R. Scown C. D. (2022). *In situ* synthesis of protic ionic liquids for biomass pretreatment. ACS Sustain. Chem. Eng. 10 (37), 12090–12098. 10.1021/acssuschemeng.2c01211

[B2] Anouti M. Caillon-Caravanier M. Le Floch C. Lemordant D. J. T. (2008). Alkylammonium-based protic ionic liquids part I: preparation and physicochemical characterization. J. Phys. Chem. B 112 (31), 9406–9411. 10.1021/jp803483f 18630861

[B3] Bahaa A. Abdelkareem M. A. Al Naqbi H. Mohamed A. Y. Yousef B. A. Sayed E. T. (2022a). Structural engineering and surface modification of nickel double hydroxide nanosheets for all-solid-state asymmetric supercapacitors. J. Energy Storage 45, 103720. 10.1016/j.est.2021.103720

[B4] Bahaa A. Abdelkareem M. A. Mohamed A. Y. Shinde P. A. Yousef B. A. Sayed E. T. (2022b). High energy storage quasi-solid-state supercapacitor enabled by metal chalcogenide nanowires and iron-based nitrogen-doped graphene nanostructures. J. Colloid Interface Sci. 608, 711–719. 10.1016/j.jcis.2021.09.136 34634546

[B5] Bahaa A. Balamurugan J. Kim N. H. Lee J. H. (2019). Metal–organic framework derived hierarchical copper cobalt sulfide nanosheet arrays for high-performance solid-state asymmetric supercapacitors. J. Mater. Chem. A Mater. 7 (14), 8620–8632. 10.1039/c9ta00265k

[B6] Balamurugan J. Nguyen T. T. Aravindan V. Kim N. H. Lee J. H. (2018). Flexible solid‐state asymmetric supercapacitors based on nitrogen‐doped graphene encapsulated ternary metal‐nitrides with ultralong cycle life. Adv. Funct. Mater. 28 (44), 1804663. 10.1002/adfm.201804663

[B7] Bard A. J. Faulkner L. R. White H. S. (2022). Electrochemical methods: fundamentals and applications. John Wiley and Sons.

[B8] Chakrabarti M. H. Mjalli F. S. AlNashef I. M. Hashim M. A. Hussain M. A. Bahadori L. (2014). Prospects of applying ionic liquids and deep eutectic solvents for renewable energy storage by means of redox flow batteries. Renew. Sustain. Energy Rev. 30, 254–270. 10.1016/j.rser.2013.10.004

[B9] Chellappan L. K. Bahaa A. Mohammed M. Bamgbopa M. O. Susantyoko R. A. (2022). Temperature-dependent electrochemical stability window of bis (trifluoromethanesulfonyl) imide and bis (fluorosulfonyl) imide anion based ionic liquids. Front. Chem. 17, 556. 10.3389/fchem.2022.859304 PMC9247390 35783210

[B10] Chen X. Y. Chen C. Zhang Z. J. Xie D. H. Deng X. Liu J. W. (2013). Nitrogen-doped porous carbon for supercapacitor with long-term electrochemical stability. J. Power Sources 230, 50–58. 10.1016/j.jpowsour.2012.12.054

[B11] Eftekhari A. Liu Y. Chen P. J. J. (2016). Different roles of ionic liquids in lithium batteries. J. Power Sources 334, 221–239. 10.1016/j.jpowsour.2016.10.025

[B12] Eftekhari AJESM (2017). Supercapacitors utilising ionic liquids. Energy Storage Mater. 9, 47–69. 10.1016/j.ensm.2017.06.009

[B13] Falk M. (2013). Determination of the dc-ion conductivity of a mixture of ionic liquids. Darmstadt, Germany: rhd instruments GmbH & Co. KG.

[B14] Filippov A. Taher M. Shah F. U. Glavatskih S. Antzutkin O. N. (2014). The effect of the cation alkyl chain length on density and diffusion in dialkylpyrrolidinium bis (mandelato) borate ionic liquids. Phys. Chem. Chem. Phys. 16 (48), 26798–26805. 10.1039/c4cp03996c 25372279

[B15] Fleshman A. M. Mauro N. A. (2019). Temperature-dependent structure and transport of ionic liquids with short-and intermediate-chain length pyrrolidinium cations. J. Mol. Liq. 279, 23–31. 10.1016/j.molliq.2019.01.108

[B16] Freire M. G. Neves C. M. Carvalho P. J. Gardas R. L. Fernandes A. M. Marrucho I. M. (2007). Mutual solubilities of water and hydrophobic ionic liquids. J. Phys. Chem. B 111 (45), 13082–13089. 10.1021/jp076271e 17958353

[B17] Giernoth R. J. (2010). Task‐specific ionic liquids. Angew. Chem. Int. Ed. Engl. 49 (16), 2834–2839. 10.1002/anie.200905981 20229544

[B18] Guo T. Zhou D. Pang L. Sun S. Zhou T. Su J. J. S. (2022). Perspectives on working voltage of aqueous supercapacitors. Small 18 (16), 2106360. 10.1002/smll.202106360 35064755

[B19] Halder P. Kundu S. Patel S. Setiawan A. Atkin R. Parthasarthy R. (2019). Progress on the pre-treatment of lignocellulosic biomass employing ionic liquids. Renew. Sustain. Energy Rev. 105, 268–292. 10.1016/j.rser.2019.01.052

[B20] Haque M. Li Q. Rigato C. Rajaras A. Smith A. D. Lundgren P. (2021). Identification of self-discharge mechanisms of ionic liquid electrolyte based supercapacitor under high-temperature operation. J. Power Sources 485, 229328. 10.1016/j.jpowsour.2020.229328

[B21] Jeong S. Li S. Appetecchi G. B. Passerini SJESM (2019). Asymmetric ammonium-based ionic liquids as electrolyte components for safer, high-energy, electrochemical storage devices. Energy Storage Mater. 18, 1–9. 10.1016/j.ensm.2019.01.015

[B22] Jing R. Jiao P. Chen J. Meng X. Wu X. Duan Y. (2021). Cas9‐Cleavage sequences in size‐reduced plasmids enhance nonviral genome targeting of CARs in primary human T cells. Small Methods 5 (7), 2100071. 10.1002/smtd.202100071 34927998

[B23] Kar M. Tutusaus O. MacFarlane D. R. Mohtadi R. J. E. Science E. (2019). Novel and versatile room temperature ionic liquids for energy storage. Energy Environ. Sci. 12 (2), 566–571. 10.1039/c8ee02437e

[B24] Lethesh K. C. Bamgbopa M. O. Susantyoko RAJF. E. R. (2021). Prospects and design insights of neat ionic liquids as supercapacitor electrolytes. Front. Energy Res. 9, 741772. 10.3389/fenrg.2021.741772

[B25] Lethesh K. C. Evjen S. Raj J. J. Roux D. C. Venkatraman V. Jayasayee K. (2019). Hydroxyl functionalized pyridinium ionic liquids: experimental and theoretical study on physicochemical and electrochemical properties. Front. Chem. 7, 625. 10.3389/fchem.2019.00625 31620423 PMC6759651

[B26] Lethesh K. C. Shah S. N. Mutalib M. I. A. (2014). Synthesis, characterization, and thermophysical properties of 1, 8-diazobicyclo [5.4. 0] undec-7-ene based thiocyanate ionic liquids. J. Chem. Eng. Data 59 (6), 1788–1795. 10.1021/je400991s

[B27] Lin R. Taberna P.-L. Fantini S. Presser V. Pérez C. R. Malbosc F. (2011). Capacitive energy storage from −50 to 100 °C using an ionic liquid electrolyte. J. Phys. Chem. Lett. 2 (19), 2396–2401. 10.1021/jz201065t

[B28] Marsousi S. Karimi-Sabet J. Moosavian M. A. Amini Y. J. (2019). Liquid-liquid extraction of calcium using ionic liquids in spiral microfluidics. Chem. Eng. J. 356, 492–505. 10.1016/j.cej.2018.09.030

[B29] Miao L. Song Z. Zhu D. Li L. Gan L. Liu M. J. E. (2021). Ionic liquids for supercapacitive energy storage: a mini-review. Energy Fuels. 35 (10), 8443–8455. 10.1021/acs.energyfuels.1c00321

[B30] Mourad E. Coustan L. Lannelongue P. Zigah D. Mehdi A. Vioux A. (2017). Biredox ionic liquids with solid-like redox density in the liquid state for high-energy supercapacitors. Nat. Mater. 16 (4), 446–453. 10.1038/nmat4808 27893725

[B31] Mousavi M. P. Dittmer A. J. Wilson B. E. Hu J. Stein A. Bühlmann P. J. J. (2015). Unbiased quantification of the electrochemical stability limits of electrolytes and ionic liquids. J. Electrochem. Soc. 162 (12), A2250–A2258. 10.1149/2.0271512jes

[B32] Muzaffar A. Ahamed M. B. Deshmukh K. Thirumalai J. J. R. (2019). A review on recent advances in hybrid supercapacitors: design, fabrication and applications. Fabr. Appl. 101, 123–145. 10.1016/j.rser.2018.10.026

[B33] Naseri F. Karimi S. Farjah E. Schaltz E. J. R. Reviews S. E. (2022). Supercapacitor management system: a comprehensive review of modeling, estimation, balancing, and protection techniques. Renew. Sustain. Energy Rev. 155, 111913. 10.1016/j.rser.2021.111913

[B34] Nasir Shah S. Shah M. U. H. Mutalib M. I. A. Lethesh K. C. Leveque J.-M. Ullah N. (2022). Ultrasonic-assisted extraction of toxic acidic components from acidic oil using 1, 8-diazobicyclo [5.4. 0] undec-7-ene-Based ionic liquids. ACS Omega 7 (31), 27479–27489. 10.1021/acsomega.2c02514 35967072 PMC9366975

[B35] Pershaanaa M. Bashir S. Ramesh S. Ramesh K. J. J. E. S. (2022). Every bite of Supercap: a brief review on construction and enhancement of supercapacitor. J. Energy Storage 50, 104599. 10.1016/j.est.2022.104599

[B36] Phakoukaki Y.-V. O'Shaughnessy P. Angeli P. J. S. Technology P. (2022). Intensified liquid-liquid extraction of biomolecules using ionic liquids in small channels. Sep. Purif. Technol. 282, 120063. 10.1016/j.seppur.2021.120063

[B37] Qi D. Liu Y. Liu Z. Zhang L. Chen X. J. (2017). Design of architectures and materials in in‐plane micro‐supercapacitors: current status and future challenges. Adv. Mater. 29 (5), 1602802. 10.1002/adma.201602802 27859675

[B38] Qi H. Ren Y. Guo S. Wang Y. Li S. Hu Y. (2019). High-voltage resistant ionic liquids for lithium-ion batteries. ACS Appl. Mater. Interfaces 12 (1), 591–600. 10.1021/acsami.9b16786 31820918

[B39] Raj J. J. Wilfred C. D. Shah S. N. Pranesh M. Mutalib M. A. Lethesh K. C. (2017). Physicochemical and thermodynamic properties of imidazolium ionic liquids with nitrile and ether dual functional groups. J. Mol. Liq. 225, 281–289. 10.1016/j.molliq.2016.11.049

[B40] Schrekker H. S. Silva D. O. Gelesky M. A. Stracke M. P. Schrekker C. M. Gonçalves R. S. (2008). Preparation, cation-anion interactions and physicochemical properties of ether-functionalized imidazolium ionic liquids. J. Braz. Chem. Soc. 19, 426–433. 10.1590/s0103-50532008000300009

[B41] Seddon K. R. Stark A. Torres MJJP (2000). Influence of chloride, water, and organic solvents on the physical properties of ionic liquids. Pure Appl. Chem. 72 (12), 2275–2287. 10.1351/pac200072122275

[B42] Shah S. N. Lethesh K. C. Mutalib M. A. Pilus R. B. Research E. C. (2015). Evaluation of thermophysical properties of imidazolium-based phenolate ionic liquids. Industrial Eng. Chem. Res. 54 (14), 3697–3705. 10.1021/ie505059g

[B43] Shah S. N. Mutalib M. A. Ismail M. F. Suleman H. Lethesh K. C. Pilus R. B. M. (2016). Thermodynamic modelling of liquid-liquid extraction of naphthenic acid from dodecane using imidazolium based phenolate ionic liquids. J. Mol. Liq. 219, 513–525. 10.1016/j.molliq.2016.03.053

[B44] Stettner T. Balducci A. J. (2021). Protic ionic liquids in energy storage devices: past, present and future perspective. Energy Storage Mater. 40, 402–414. 10.1016/j.ensm.2021.04.036

[B45] Susantyoko R. A. Karam Z. Alkhoori S. Mustafa I. Wu C. H. Almheiri S. J. J. (2017). A surface-engineered tape-casting fabrication technique toward the commercialisation of freestanding carbon nanotube sheets. J. Mater. Chem. A Mater. 5 (36), 19255–19266. 10.1039/c7ta04999d

[B46] Timperman L. Skowron P. Boisset A. Galiano H. Lemordant D. Frackowiak E. (2012). Triethylammonium bis (tetrafluoromethylsulfonyl) amide protic ionic liquid as an electrolyte for electrical double-layer capacitors. Phys. Chem. Chem. Phys. 14 (22), 8199–8207. 10.1039/c2cp40315c 22546714

[B47] Venkatraman V. Lethesh K. C. (2019). Establishing predictive models for solvatochromic parameters of ionic liquids. Front. Chem. 7, 605. 10.3389/fchem.2019.00605 31552223 PMC6733962

[B48] Wang R. Fang C. Yang L. Li K. Zhu K. Liu G. (2022). The novel ionic liquid and its related self‐assembly in the areas of energy storage and conversion. Small Sci. 2 (9), 2200048. 10.1002/smsc.202200048 PMC11935792 40212637

[B49] Wang X. Chi Y. Mu T. J. J. (2014). A review on the transport properties of ionic liquids. J. Mol. Liq. 193, 262–266. 10.1016/j.molliq.2014.03.011

[B50] Watanabe M. Thomas M. L. Zhang S. Ueno K. Yasuda T. Dokko K. (2017). Application of ionic liquids to energy storage and conversion materials and devices. Chem. Rev. 117 (10), 7190–7239. 10.1021/acs.chemrev.6b00504 28084733

