# Supplementary material for: Ionic liquid electrolyte selection for high voltage supercapacitors in high-temperature applications
Source: Front Chem. 2024 Mar 4;12:1349864. doi: 10.3389/fchem.2024.1349864 (PMC10945003; doi:10.3389/fchem.2024.1349864)
Supplement: Supplementary file 1 [file DataSheet1.DOCX]

**Supporting information**

**Ionic Liquid Electrolyte Screening for High Voltage Supercapacitors in High-Temperature Applications**

Ahmed Bahaa, Ayoob Alhammadi, Kallidanthiyil Chellappan Lethesh, Rahmat Agung Susantyoko, Musbaudeen O. Bamgbopa^*^

R&D Centre, Dubai Electricity and Water Authority (DEWA), P O Box-564, Dubai, United Arab Emirates

* Corresponding author Tel.: +971 4 515 0257

E-mail: [musbaudeen.bamgbopa@dewa.gov.ae](mailto:musbaudeen.bamgbopa@dewa.gov.ae) (M. O. Bamgbopa)

**Table S1.** Nomenclature, catalogue details, and ESW of the ILs at room temperature (RT) utilized in this study.

| **Entry** | **ILs** | **Abbreviation** | **CAS Number** | **ESW**(1) **(V)** |
| --- | --- | --- | --- | --- |
| 1 | 1-Ethyl-3-methylimidazolium bis(trifluoromethanesulfonyl)imide | [EMIM] [TFSI] | 174899-82-2 | 4.3 |
| 2 | 1-Ethyl-3-methylimidazolium bis(fluorosulfonyl)imide | [EMIM] [FSI] | 235789-75-0 | 4.5 |
| 3 | N-Propyl-N-methylpyrrolidinium bis(trifluoromethanesulfonyl)imide | [Pyr _1, 3_] [TFSI] | 223437-05-6 | 6 |
| 4 | N-Propyl-N-methylpyrrolidinium bis(fluorosulfonyl)imide | [Pyr _1, 3_] [FSI] | 852620-97-4 | 5.8 |
| 5 | N-Propyl-N-methylpyrrolidinium bis(trifluoromethanesulfonyl)imide | [Pyr _1, 4_] [TFSI] | 223437-11-4 | 5.9 |
| 6 | 1-Butyl-1-methylpyrrolidinium bis(fluorosulfonyl)imide | [Pyr _1, 4_] [FSI] | 1057745-51-3 | 5.9 |
| 7 | N-Pentyl-N-methylpyrrolidinium bis(trifluoromethanesulfonyl)imide | [Pyr _1, 5_] [TFSI] | 223437-05-6 | 5.9 |
| 8 | 1-Methyl-1-(2-methoxyethyl)pyrrolidinium Bis(trifluoromethanesulfonyl)imide | [Pyr _1, 102_] [TFSI] | 757240-24-7 | 5.8 |
| 9 | 1-Methyl-1-(2-methoxypropyl)pyrrolidinium Bis(trifluoromethanesulfonyl)imide | [Pyr _1, 103_] [TFSI] | - | 4.9 |
| 10 | 1-Methyl-1-(2-methoxypropyl)pyrrolidinium bis(fluorosulfonyl)imide | [Pyr _1, 103_] [FSI] | - | 4.3 |
| 11 | N-butyl-N-methylpiperidinium bis(trifluoromethanesulfonyl)imide | [Pip _1, 4_] [TFSI] | 623580-02-9 | 6 |
| 12 | 1-Methyl-1-propylpiperidinium bis(trifluoromethanesulfonyl)imide | [Pip_1, 3_] [TFSI] | 608140-12-1 | 6 |
| 13 | 1-Methyl-1-propylpiperidinium bis(fluorosulfonyl)imide | [Pip_1, 3_] [FSI] | 911303-46-3 | 5.7 |
| 14 | N-Trimethyl-N-propylammonium bis(trifluoromethanesulfonyl)imide | [N _111, 3_] [TFSI] | 268536-05-6 | 5.9 |
| 15 | N-Trimethyl-N-butylammonium bis(trifluoromethanesulfonyl)imide | [N _111, 4_] [TFSI] | 258273-75-5 | 5.7 |
| 16 | N-Trimethyl-N-hexylammonium bis(trifluoromethanesulfonyl)imide | [N _111, 6_] [TFSI] | 210230-43-6 | 5.8 |
| 17 | N-Tributyl-N-methylammonium bis(trifluoromethanesulfonyl)imide, | [N _444, 1_] [TFSI] | 405514-94-5 | 5.9 |
| 18 | N-ethyl-N,N-dimethyl-N(2methoxyethyl)ammonium bis(trifluoromethylsulfonyl)imide | [N _112, 102_] [TFSI] | 557788-37-1 | 4.8 |
| 19 | N-ethyl-N,N-dimethyl-N(2methoxyethyl)ammonium bis(fluorosulfonyl)imide | [N _112, 102_] [FSI] | 1235234-35-1 | 5.9 |
| 20 | N,N-diethyl-N-methyl-N-(2-methoxyethyl)ammonium bis(trifluoromethanesulfonyl)imide | [N _221, 102_] [FSI] | 557788-37-1 | 5.9 |
| 21 | N,N-Dimethyl-N-Ethyl-N-Phenylethylammonium Bis(trifluoromethanesulfonyl)imide | [N _112_, PhenylEth] [TFSI] | - | 4.2 |
| 22 | N,N-Dimethyl-N-ethyl-N-benzylAmmonium Bis(trifluoromethanesulfonyl)imide | [N _112_, Benz] [TFSI] | - | 4.6 |

**
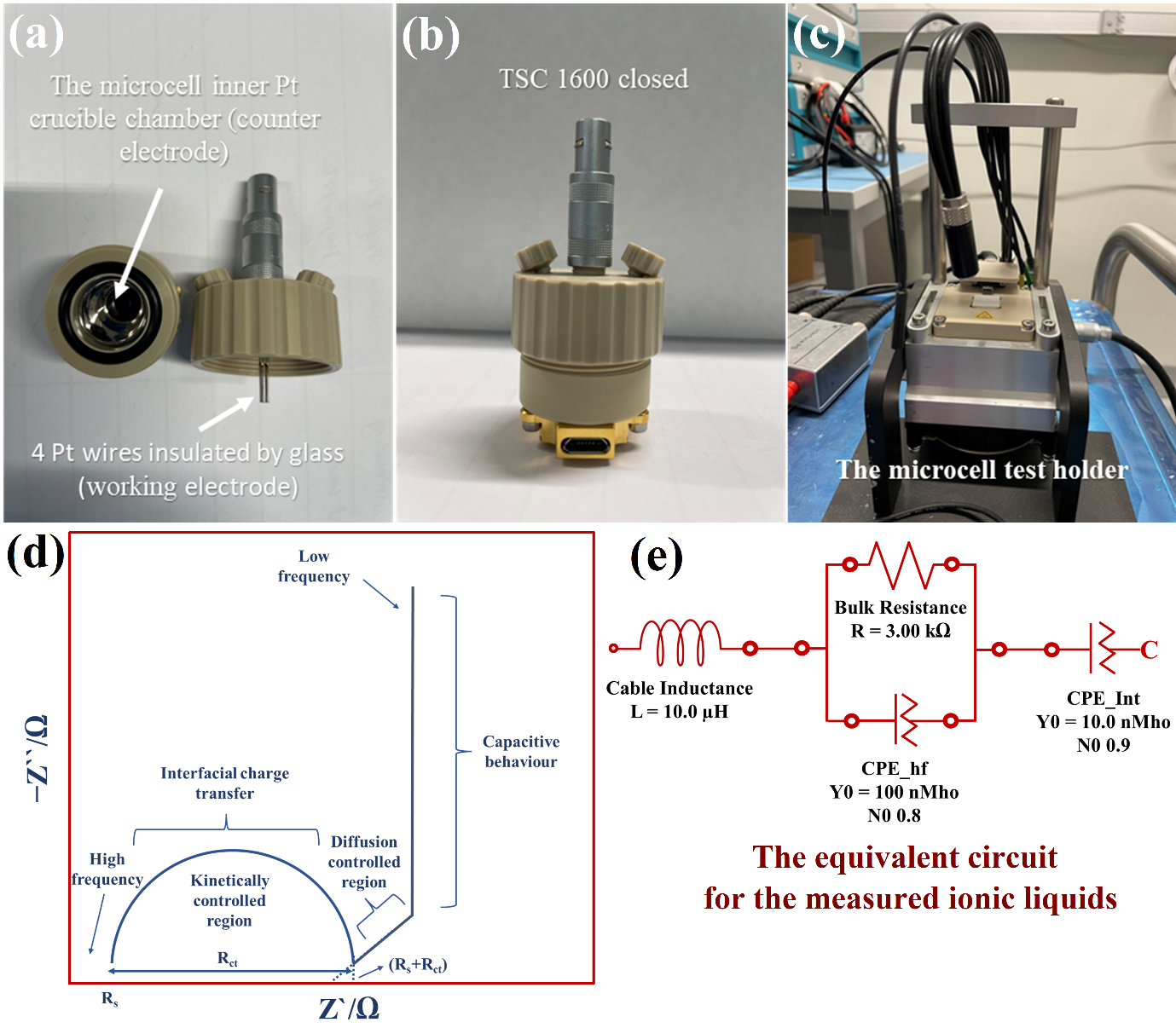
**

Figure S1. a) The microcell inner chamber, b) the microcell after assembly, c) the test station holder, d) The EIS measurement with the real and imaginary parts,(2) and e) the equivalent circuit for the measured EIS.


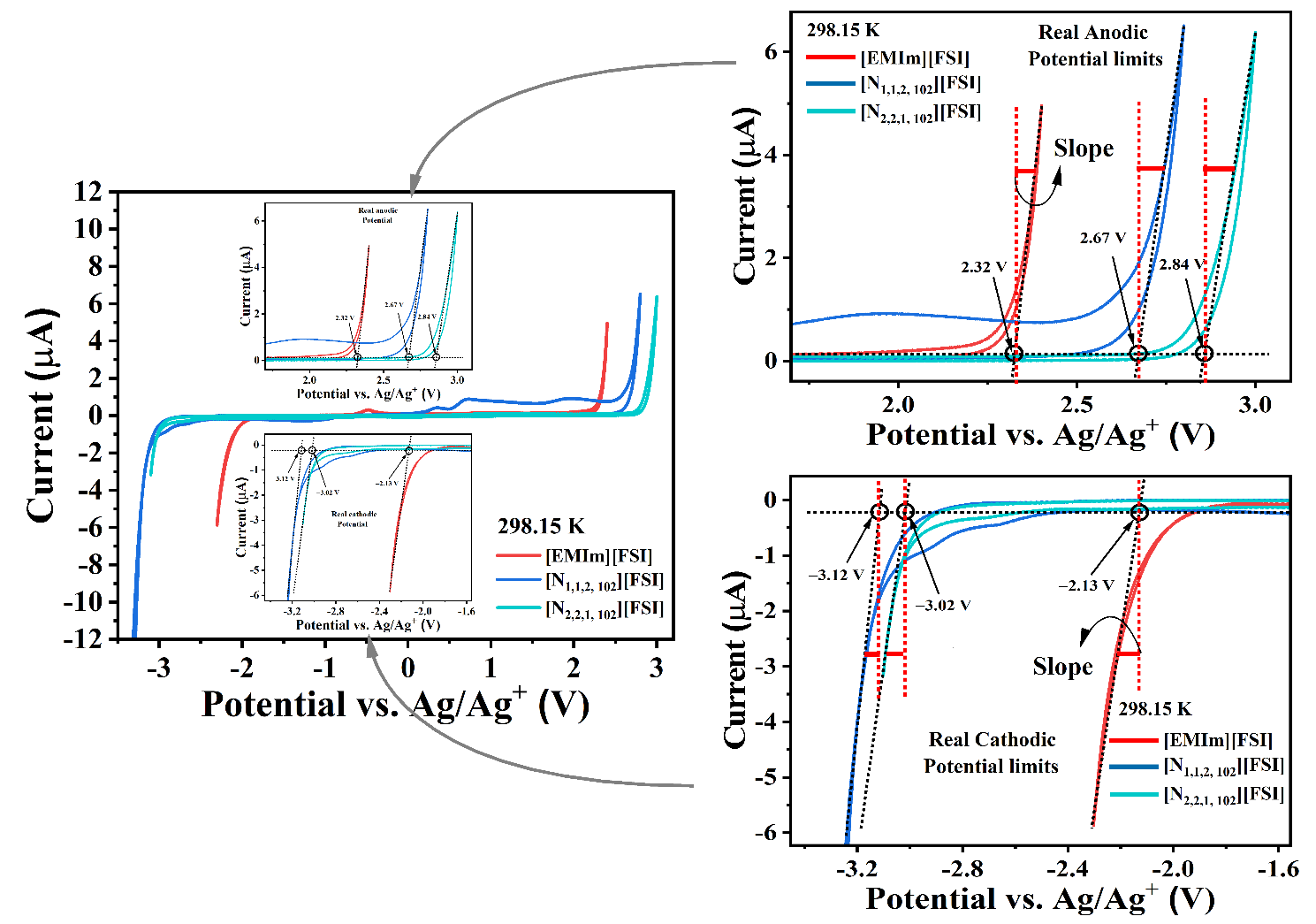


**Figure S2.** Sample CV measurements of FSI-anion based ILs. Effect of mass transport on electrochemical stability window depicted with tangents and slopes, and it`s role in selecting optimum ILs for practical applications.

**Table S2.** Density (ρ, g.cm^−3^) of [FSI] anion based ILs.

| **T(K)** | **[Pip _1, 3_] [FSI]** | **[Pyr _1, 3_] [FSI]** |
| --- | --- | --- |
| 278.15 | 1.3368 | 1.3556 |
| 283.15 | 1.3325 | 1.3511 |
| 288.15 | 1.3299 | 1.3470 |
| 293.15 | 1.3299 | 1.3429 |
| 298.15 | 1.3297 | 1.3388 |
| 303.15 | 1.3173 | 1.3348 |
| 308.15 | 1.3133 | 1.3309 |
| 313.15 | 1.3095 | 1.3270 |
| 318.15 | 1.3057 | 1.3231 |
| 323.15 | 1.3019 | 1.3192 |
| 328.15 | 1.2981 | 1.3153 |
| 333.15 | 1.2944 | 1.3114 |
| 338.15 | 1.2908 | 1.3076 |
| 343.15 | 1.2870 | 1.3038 |
| 348.15 | 1.2834 | 1.3000 |
| 353.15 | 1.2798 | 1.2962 |
| 358.15 | 1.2761 | 1.2925 |
| 363.15 | 1.2725 | 1.2887 |
| 368.15 | 1.2690 | 1.2850 |
| 373.15 | 1.2654 | 1.2813 |

Table S3. Density (ρ, g.cm^−3^) of [TFSI] anion based ILs.

| **T/K** | **[Pyr _1, 102_]**  **[TFSI]** | **[Pip _1, 3_]**  **[TFSI]** | **[Pyr _1, 3_]**  **[TFSI]** | **[Pyr _1, 5_]**  **[TFSI]** | **[N _111, 3_]**  **[TFSI]** | **[N _111, 6_]**  **[TFSI]** |
| --- | --- | --- | --- | --- | --- | --- |
| 278.15 | 1.4729 | 1.4276 | 1.4462 | 1.3788 | 1.4447 | 1.3468 |
| 283.15 | 1.4677 | 1.4230 | 1.4413 | 1.3743 | 1.4397 | 1.3423 |
| 288.15 | 1.4629 | 1.4185 | 1.4367 | 1.3699 | 1.4349 | 1.3379 |
| 293.15 | 1.4581 | 1.4141 | 1.4321 | 1.3654 | 1.4302 | 1.3336 |
| 298.15 | 1.4533 | 1.4098 | 1.4275 | 1.3611 | 1.4256 | 1.3293 |
| 303.15 | 1.4486 | 1.4053 | 1.4230 | 1.3567 | 1.4208 | 1.3249 |
| 308.15 | 1.4439 | 1.4010 | 1.4185 | 1.3524 | 1.4162 | 1.3206 |
| 313.15 | 1.4392 | 1.3965 | 1.4140 | 1.3481 | 1.4116 | 1.3163 |
| 318.15 | 1.4346 | 1.3922 | 1.4095 | 1.3438 | 1.4069 | 1.31201 |
| 323.15 | 1.4299 | 1.3881 | 1.4051 | 1.3395 | 1.4023 | 1.3075 |
| 328.15 | 1.4253 | 1.3835 | 1.4007 | 1.3353 | 1.3977 | 1.3031 |
| 333.15 | 1.4207 | 1.3792 | 1.3963 | 1.3311 | 1.3931 | 1.2987 |
| 338.15 | 1.4162 | 1.3750 | 1.3919 | 1.3268 | 1.3885 | 1.2943 |
| 343.15 | 1.4116 | 1.3707 | 1.3875 | 1.3227 | 1.3838 | 1.2898 |
| 348.15 | 1.4071 | 1.3665 | 1.3832 | 1.3185 | 1.3792 | 1.2852 |
| 353.15 | 1.4026 | 1.3623 | 1.3789 | 1.3143 | 1.3745 | 1.2805 |
| 358.15 | 1.3981 | 1.3581 | 1.3746 | 1.3102 | 1.3699 | 1.2757 |
| 363.15 | 1.3937 | 1.3539 | 1.3703 | 1.3060 | 1.3651 | 1.2707 |
| 368.15 | 1.3892 | 1.3497 | 1.3660 | 1.3019 | 1.3604 | 1.265 |
| 373.15 | 1.3848 | 1.3457 | 1.3618 | 1.2978 | 1.3557 | 1.2603 |

Table S4. Comparison of density and viscosity of selected ILs with literature data at room temperature.

| **Entry** |  | **ILs** | **Density** | **Reference** | **Dynamic Viscosity** | **Reference** |
| --- | --- | --- | --- | --- | --- | --- |
| 1 |  | [Pyr _1, 3_] [FSI] | 1.339  1.338  1.338 | [(3)]  [(4)]  This Work | 40.60  39.80  41.36 | [(3)]  [(5)]  This work |
| 2 |  | [Pyr _1, 3_] [TFSI] | 1.429  1.428  1.427 | [(6)]  [(7)]  This Work | 61.00  54.00  61.19 | [(8)]  [(9)]  This work |
| 3 |  | [Pip _1, 3_] [TFSI] | 1.409  1.409  1.409 | [(10)]  [(8)]  This Work | 147.03  145.60  147.01 | [(8)]  [(11)]  This work |
| 4 |  | [Pip _1, 3_] [FSI] | 1.320  1.320  1.321 | [(4)]  [(12)]  This Work | 51.00  92.17 | [(8)]  This work |
|  |  |  |  |  |  |  |

**Table S5.** Dynamic viscosity (ղ, mPa.s) of [FSI] anion based ILs.

| **T(K)** | **[Pip _1, 3_] [FSI]** | **[Pyr _1, 3_] [FSI]** |
| --- | --- | --- |
| 278.15 | 247.38 | 85.53 |
| 283.15 | 188.69 | 70.26 |
| 288.15 | 146.10 | 58.26 |
| 293.15 | 115.11 | 48.84 |
| 298.15 | 92.17 | 41.36 |
| 303.15 | 74.90 | 35.36 |
| 308.15 | 61.68 | 30.50 |
| 313.15 | 51.40 | 26.52 |
| 318.15 | 43.30 | 23.23 |
| 323.15 | 36.86 | 20.48 |
| 328.15 | 31.65 | 18.17 |
| 333.15 | 27.41 | 16.22 |
| 338.15 | 23.92 | 14.55 |
| 343.15 | 21.02 | 13.12 |
| 348.15 | 18.59 | 11.89 |
| 353.15 | 16.55 | 10.82 |
| 358.15 | 14.81 | 9.89 |
| 363.15 | 13.32 | 9.07 |
| 368.15 | 12.05 | 8.35 |
| 373.15 | 10.95 | 7.72 |

**Table S6.** Dynamic viscosity (ղ, mPa.s) of [TFSI] anion based ILs.

| **T(K)** | **[Pyr _1, 102_] [TFSI]** | **[Pip _1, 3_]**  **[TFSI]** | **[Pyr _1, 3_]**  **[TFSI]** | **[Pyr _1, 5_]**  **[TFSI]** | **[N _111, 3_]**  **[TFSI]** | **[N _111, 6_]**  **[TFSI]** |
| --- | --- | --- | --- | --- | --- | --- |
| 278.15 | 146.39 | 580.59 | 165.79 | 305.38 | 239.96 | 592.67 |
| 283.15 | 111.25 | 393.61 | 125.81 | 222.62 | 175.96 | 411.56 |
| 288.15 | 86.106 | 275.29 | 97.14 | 165.53 | 131.89 | 292.44 |
| 293.15 | 67.996 | 198.53 | 76.42 | 125.7 | 101.01 | 212.91 |
| 298.15 | 54.616 | 147.01 | 61.19 | 97.38 | 78.92 | 158.75 |
| 303.15 | 44.569 | 111.52 | 49.76 | 76.81 | 62.84 | 120.96 |
| 308.15 | 36.900 | 86.40 | 41.05 | 61.58 | 50.85 | 93.98 |
| 313.15 | 30.923 | 68.24 | 34.31 | 50.10 | 41.76 | 74.31 |
| 318.15 | 26.226 | 54.82 | 29.00 | 41.31 | 34.76 | 59.67 |
| 323.15 | 22.459 | 44.72 | 24.77 | 34.48 | 29.29 | 48.63 |
| 328.15 | 19.420 | 36.98 | 21.36 | 29.11 | 24.89 | 40.14 |
| 333.15 | 16.938 | 30.98 | 18.58 | 24.82 | 21.39 | 33.53 |
| 338.15 | 14.881 | 26.24 | 16.29 | 21.36 | 18.55 | 28.31 |
| 343.15 | 13.170 | 22.45 | 14.38 | 18.54 | 16.22 | 24.15 |
| 348.15 | 11.731 | 19.40 | 12.79 | 16.21 | 14.29 | 20.78 |
| 353.15 | 10.514 | 16.90 | 11.44 | 14.29 | 12.68 | 18.04 |
| 358.15 | 9.4756 | 14.84 | 10.29 | 12.67 | 11.32 | 15.78 |
| 363.15 | 8.5857 | 13.13 | 9.31 | 11.31 | 10.16 | 13.90 |
| 368.15 | 7.8199 | 11.69 | 8.47 | 10.16 | 9.180 | 12.33 |
| 373.15 | 7.1567 | 10.48 | 7.74 | 9.17 | 8.33 | 11.01 |

**Table S7.** Dc-ionic conductivity (σ, mScm^−1^) of selected 8 ILs at different temperature ranges.

| **T/K** | **[Pyr _1, 3_] [TFSI]** | **[Pyr _1, 5_] [TFSI]** | **[Pyr _1, 3_] [FSI]** | **[Pyr _1, 102_] [TFSI]** | **[Pip _1, 3_] [TFSI]** | **[Pip _1, 3_] [FSI]** | **[N _111, 3_] [TFSI]** | **[N _111, 6_] [TFSI]** |
| --- | --- | --- | --- | --- | --- | --- | --- | --- |
| 278.15 | 2.249 | 1.005 | 6.346 | 2.125 | 0.809 | 2.166 | 1.592 | 0.551 |
| 298.15 | 3.212 | 1.507 | 7.658 | 3.041 | 1.284 | 3.107 | 2.470 | 0.853 |
| 313.15 | 4.983 | 2.537 | 10.162 | 4.685 | 2.271 | 4.827 | 4.028 | 1.518 |
| 333.15 | 8.172 | 4.519 | 14.824 | 7.757 | 4.316 | 8.083 | 6.848 | 2.905 |
| 358.15 | 13.482 | 7.063 | 21.488 | 12.489 | 8.196 | 13.570 | 11.800 | 5.428 |

**Table S8.** The details of curve fits of density ($\rho$) as a function of temperature (T) as per the equation $\rho=A+BT+CT^{2}$. The accuracy of fit was measured using R^2^, where the closer the value of R^2^ to 1, the better the fit. The fitting was performed based on empirical correlation in reference [13].

| **ILs** | ***A* (g cm^-3^)** | ***B* (g cm^-3^ K^-1^)** | ***C* (g cm^-3^ K^-2^)** | **R^2^** |
| --- | --- | --- | --- | --- |
| **[Pip _1, 3_] [FSI]** | 1.59186 | -0.00104 | 4.46708E-7 | 0.99999 |
| **[Pyr _1, 3_] [FSI]** | 1.62398 | -0.00111 | 5.04967E-7 | 0.99998 |
| **[Pyr _1, 102_] [TFSI]** | 1.78284 | -0.00126 | 5.12281E-7 | 0.99999 |
| **[Pip _1, 3_] [TFSI]** | 1.70629 | -0.00111 | 3.75256E-7 | 0.99999 |
| **[Pyr _1, 3_] [TFSI]** | 1.74085 | -0.00119 | 4.66667E-7 | 0.99999 |
| **[Pyr _1, 5_] [TFSI]** | 1.65773 | -0.00112 | 4.05559E-7 | 1 |
| **[N _111, 3_] [TFSI]** | 1.70832 | -9.61899E-4 | 4.689E-8 | 0.99998 |
| **[N _111, 6_] [TFSI]** | 1.51372 | -3.81066E-4 | -7.93643E-7 | 0.99988 |

**Table S9.** The details of curve fits of viscosity ($\eta$) as a function of temperature (T) using the classical Vogel−Fulcher−Tammann (VFT) equation $\eta=\eta_{0} exp \left( \frac{D}{\left( T-T_{0} \right)} \right)$. The accuracy of fit was measured using R^2^, where the closer the value of R^2^ to 1, the better the fit. The fitting was performed based on empirical correlation in reference [13].

| **ILs** | $\boldsymbol{\eta}_{\boldsymbol{0}}$ ***(mPa.s)*** | ***D (K)*** | $\boldsymbol{T}_{\boldsymbol{0}}$ ***(K)*** | **R^2^** |
| --- | --- | --- | --- | --- |
| **[Pip _1, 3_] [FSI]** | 0.152740021 | 958.6843592 | 148.443 | 1 |
| **[Pyr _1, 3_] [FSI]** | 0.156973883 | 966.3781358 | 124.78832 | 1 |
| **[Pyr _1, 102_] [TFSI]** | 0.173553809 | 786.4815761 | 161.43028 | 1 |
| **[Pip _1, 3_] [TFSI]** | 0.133881198 | 855.0061838 | 176.06438 | 1 |
| **[Pyr _1, 3_] [TFSI]** | 0.161522072 | 826.3377551 | 158.99351 | 0.99999 |
| **[Pyr _1, 5_] [TFSI]** | 0.107618201 | 947.9241583 | 158.94452 | 0.99999 |
| **[N _111, 3_] [TFSI]** | 0.149177751 | 833.9104998 | 165.21445 | 1 |
| **[N _111, 6_] [TFSI]** | 0.078226051 | 1035.415921 | 162.25491 | 0.99999 |

**Table S10.** The details of curve fits of conductivity ($\sigma$) as a function of temperature (T) using the equation $\sigma=\sigma_{0}exp\left( \frac{E}{\left( T_{0}-T \right)} \right)$. The accuracy of fit was measured using R^2^, where the closer the value of R^2^ to 1, the better the fit. The fitting was performed based on empirical correlation in reference [13].

| **ILs** | $\boldsymbol{\sigma}_{\mathbf{0}}$ **(mS cm^-1^)** | $\boldsymbol{E}$ **(K)** | $\mathbf{T}_{\mathbf{0}}$ **(K)** | **R^2^** |
| --- | --- | --- | --- | --- |
| **[Pip _1, 3_] [FSI]** | 1293.72192 | 1113.56605 | 113.80238 | 1 |
| **[Pyr _1, 3_] [FSI]** | 3942.18744 | 1898.96692 | -6.3192 | 0.99998 |
| **[Pyr _1, 102_] [TFSI]** | 457.3933 | 763.36637 | 146.11048 | 1 |
| **[Pip _1, 3_] [TFSI]** | 1493.11274 | 1185.76369 | 130.33587 | 1 |
| **[Pyr _1, 3_] [TFSI]** | 882.44605 | 978.99279 | 124.02313 | 1 |
| **[Pyr _1, 5_] [TFSI]** | 78.27992 | 369.50592 | 204.35596 | 0.99999 |
| **[N _111, 3_] [TFSI]** | 729.63542 | 893.44494 | 141.566 | 1 |
| **[N _111, 6_] [TFSI]** | 654.07479 | 1032.26349 | 142.70394 | 1 |

**Table S11.** The details of curve fits of Walden plots of molar conductivity σ and viscosity η for ILs. The solid lines are correlated with equation log σ = log C′ + α log η^-1^. The accuracy of fit was measured using R^2^, where the closer the value of R^2^ to 1, the better the fit. The fitting was performed based on empirical correlation in reference [13].

| **ILs** | **log C′ (S cm^2^ mol^-1^)** | **α** | **R^2^** |
| --- | --- | --- | --- |
| **[Pip _1, 3_] [FSI]** | -0.9705 | 0.8186 | 0.9995 |
| **[Pyr _1, 3_] [FSI]** | -0.7397 | 0.7175 | 0.9961 |
| **[Pyr _1, 102_] [TFSI]** | -1.0915 | 0.8228 | 0.9996 |
| **[Pip _1, 3_] [TFSI]** | -1.0833 | 0.8052 | 0.9986 |
| **[Pyr _1, 3_] [TFSI]** | -1.0258 | 0.8148 | 0.9994 |
| **[Pyr _1, 5_] [TFSI]** | -1.1064 | 0.7852 | 0.9991 |
| **[N _111, 3_] [TFSI]** | -1.0989 | 0.8270 | 0.9996 |
| **[N _111, 6_] [TFSI]** | -1.1996 | 0.8015 | 0.999 |

**Reference**

1. Chellappan LK, Bahaa A, Mohammed M, Bamgbopa MO, Susantyoko RAJFiC. Temperature-dependent electrochemical stability window of bis (trifluoromethanesulfonyl) imide and bis (fluorosulfonyl) imide anion based ionic liquids. 2022:556.

2. Kandula S, Shrestha KR, Kim NH, Lee JHJS. Fabrication of a 3D hierarchical sandwich Co9S8/α‐MnS@ N–C@ MoS2 nanowire architectures as advanced electrode material for high performance hybrid supercapacitors. 2018;14(23):1800291.

3. Yamamoto T, Matsumoto K, Hagiwara R, Nohira TJTJoPCC. Physicochemical and electrochemical properties of K [N (SO2F) 2]–[N-methyl-N-propylpyrrolidinium][N (SO2F) 2] ionic liquids for potassium-ion batteries. 2017;121(34):18450-8.

4. Rabhi F, Hussard C, Sifaoui H, Mutelet FJJoML. Characterization of bis (fluorosulfonyl) imide based ionic liquids by gas chromatography. 2019;289:111169.

5. Huang Q, Lee Y-Y, Gurkan BJI, Research EC. Pyrrolidinium ionic liquid electrolyte with bis (trifluoromethylsulfonyl) imide and bis (fluorosulfonyl) imide anions: lithium solvation and mobility, and performance in lithium metal–lithium iron phosphate batteries. 2019;58(50):22587-97.

6. Musiał M, Zorębski E, Zorębski M, Dzida MJJoML. Effect of alkyl chain length in cation on thermophysical properties of two homologous series: 1-alkyl-1-methylpyrrolidinium bis (trifluoromethylsulfonyl) imides and 1-alkyl-3-methylimidazolium trifluoromethanesulfonates. 2019;293:111511.

7. Zorębski E, Zorębski M, Musiał M, Dzida MJTJoPCB. Ultrasonic relaxation spectra for pyrrolidinium bis (trifluoromethylsulfonyl) imides: A comparison with imidazolium bis (trifluoromethylsulfonyl) imides. 2017;121(42):9886-94.

8. Seki S, Serizawa N, Ono S, Takei K, Hayamizu K, Tsuzuki S, et al. Densities, Viscosities, and Refractive Indices of binary room-temperature ionic liquids with common cations/anions. 2019;64(2):433-41.

9. Jin H, O'Hare B, Dong J, Arzhantsev S, Baker GA, Wishart JF, et al. Physical properties of ionic liquids consisting of the 1-butyl-3-methylimidazolium cation with various anions and the bis (trifluoromethylsulfonyl) imide anion with various cations. 2008;112(1):81-92.

10. Paduszyński K, Chiyen J, Ramjugernath D, Letcher TM, Domańska UJFPE. Liquid–liquid phase equilibrium of (piperidinium-based ionic liquid+ an alcohol) binary systems and modelling with NRHB and PCP-SAFT. 2011;305(1):43-52.

11. Bhattacharjee A, Carvalho PJ, Coutinho JAJFPE. The effect of the cation aromaticity upon the thermophysical properties of piperidinium-and pyridinium-based ionic liquids. 2014;375:80-8.

12. Nath D, Sumon KZ, Tagiuri A, Henni AJFPE. Effect of cation on the solubility of ethane in three bis (fluorosulfonyl) imide ([FSI]) based low viscosity ionic liquids. 2017;454:78-90.

13. Y. Zheng, Y. Zheng, Q. Wang, and Z. Wang, Density, Viscosity, and Electrical Conductivity of 1-Alkyl-3-methylimidazolium Dicyanamide Ionic Liquids. J. Chem. Eng. Data, vol. 66, no. 1, pp. 480–493, Jan. 2021, doi: 10.1021/acs.jced.0c00754.
